# The metabolic-inflammatory axis in chronic heart failure: integrating the NLR and TyG index for recent-onset atrial fibrillation prediction via machine learning and mediation analysis

**DOI:** 10.3389/fendo.2026.1831904

**Published:** 2026-08-26

**Authors:** Chenglong Yao, Xinmei Liu, Runjia Liu, Dongdong Su, Kai Yang, Ling Yao, Hongfan Qiu, Bingjie Wang, Xuemei Hou, Jianming Yao, Yuerong Jiang, Haixia Li

**Affiliations:** 1 Guang’anmen Hospital, China Academy of Chinese Medical Sciences, Beijing, China; 2 Shandong University of Traditional Chinese Medicine, Jinan, China; 3 Jinan Municipal Hospital of Traditional Chinese Medicine, Jinan, Shandong, China; 4 Xiyuan Hospital, China Academy of Chinese Medical Sciences, Beijing, China

**Keywords:** chronic heart failure, machine learning, neutrophil-to-lymphocyte ratio, recent-onset atrial fibrillation, TyG index

## Abstract

**Introduction:**

Recent-onset atrial fibrillation (AF) is a common complication of chronic heart failure (CHF), potentially involving both inflammatory and metabolic dysregulation. This study aimed to develop and externally validate an interpretable machine learning (ML) model for predicting recent-onset AF in patients with CHF and to explore the relationships among inflammatory dysregulation, metabolic dysregulation, and recent-onset AF.

**Methods:**

In this retrospective multicenter study, 4,872 hospitalized patients with CHF from Guang’anmen Hospital and Xiyuan Hospital were included, with external validation performed in 277 additional patients. Demographic, clinical, and laboratory variables, including the triglyceride-glucose (TyG) index and neutrophil-to-lymphocyte ratio (NLR), were analyzed. Nine ML algorithms were compared for AF prediction. Model interpretability was assessed using SHapley Additive exPlanations (SHAP). Segmented regression was used to examine nonlinear threshold effects, and mediation analysis was performed to explore the immunometabolic pathway linking TyG, NLR, and AF.

**Results:**

The Extra Trees model performed best, achieving an area under the receiver operating characteristic curve of 0.853 in the training cohort and 0.766 in the external validation cohort. NLR showed a nonlinear association with AF risk, with a steeper increase below 4.24, whereas TyG showed a threshold-dependent J-shaped relationship, with the risk increasing significantly above 5.91. The combination of TyG and NLR further improved model discrimination. Mediation analysis suggested that the estimated indirect association through NLR accounted for a substantial proportion of the observed association between TyG and AF.

**Conclusions:**

An interpretable ML framework identified the immune-metabolic axis as an important predictive component of recent-onset AF in CHF. NLR and TyG are inexpensive, clinically accessible biomarkers that may improve early risk stratification and identify a high-risk phenotype characterized by concurrent metabolic stress and inflammation. Trial registration: ChiCTR (ITMCTR2025001576).

## Graphical Abstract

**Figure:**
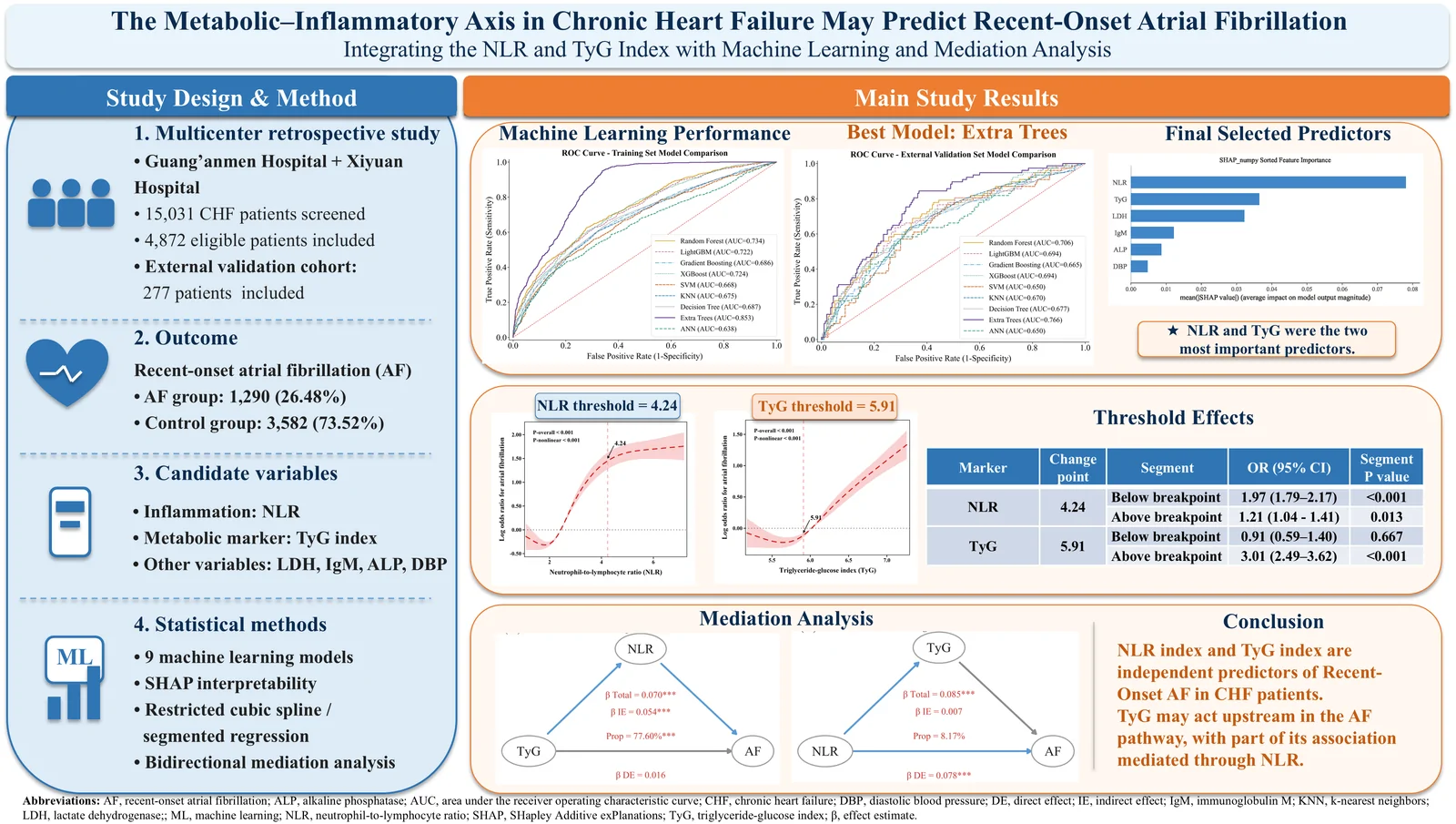

## 1 Introduction

Heart failure (HF) is a complex clinical syndrome caused by structural and/or functional cardiac abnormalities that impair ventricular filling or ejection, resulting in elevated intracardiac pressure, reduced cardiac output, or both (1, 2). As a major global public health challenge, HF affects an estimated 64 million people worldwide and is associated with substantial morbidity, mortality, recurrent hospitalization, and healthcare expenditure (3). Chronic heart failure (CHF), the most common long-term phenotype of HF, is characterized by progressive myocardial remodeling, recurrent decompensation, and persistent symptoms, with an increasing prevalence in aging populations. Atrial fibrillation (AF) is a common and clinically important comorbidity associated with poorer hemodynamic status, higher risks of rehospitalization and death, and greater management complexity. Their relationship is bidirectional: AF aggravates ventricular dysfunction by impairing atrial contraction and diastolic filling and causing rapid or irregular ventricular responses, whereas CHF promotes AF through atrial stretch, fibrosis, electrical remodeling, neurohormonal activation, inflammation, oxidative stress, and metabolic disturbance (4, 5). Given the multifactorial and progressive nature of these mechanisms, conventional single clinical indicators may be insufficient for identifying CHF patients at high risk of AF.

Routine laboratory biomarkers reflecting inflammation, immune imbalance, and metabolic dysfunction may provide a scalable and low-cost strategy for risk assessment. Among these markers, the neutrophil-to-lymphocyte ratio (NLR) is an accessible composite indicator of innate immune activation and relative adaptive immune suppression (6); the systemic immune-inflammation index (SII) (7), platelet-to-lymphocyte ratio (PLR) (8), and systemic inflammation response index (SIRI) summarize related inflammatory states (9); and the triglyceride-glucose (TyG) index serves as a surrogate marker of insulin resistance and metabolic stress (10). Although these biomarkers have each been linked to adverse cardiovascular outcomes, they have rarely been integrated into interpretable prediction models for recent-onset AF in CHF. Moreover, most previous studies focused on association rather than clinically usable prediction, and few explored the possible interplay between metabolic dysregulation and inflammation.

Our study was designed to develop and validate an interpretable machine learning (ML) model for predicting the risk of recent-onset AF in patients with chronic heart failure (CHF) on the basis of routinely collected clinical features and biomarkers associated with systemic immune inflammation. This retrospective study included patients with CHF. Using routinely assessed clinical indicators and biological mechanisms associated with AF in HF patients, we incorporated biomarkers related to immunity and inflammation. Nine ML models were used to evaluate the associations of these biomarkers with the risk of recent-onset AF in patients with chronic heart failure. Thereafter, the best-performing machine learning model for predicting AF onset in patients with chronic heart failure was selected. We further conducted mediation analyses to examine the statistical relationships among TyG, NLR, and recent-onset AF in patients with CHF.

## 2 Materials and methods

### 2.1 Study design and population

This multicenter retrospective study enrolled consecutive patients with CHF from the Department of Cardiology of Guang’anmen Hospital, China Academy of Chinese Medical Sciences, and Xiyuan Hospital, China Academy of Chinese Medical Sciences, between January 2015 and December 2024. An independent cohort from Jinan Municipal Hospital of Traditional Chinese Medicine was used for external validation.

All patients in this study were informed and provided written informed consent. The study protocol was published and registered at www.chictr.org.cn (Registration No. ITMCTR2025001576). The study protocol was approved by the Medical Ethics Committee of Guang’anmen Hospital, China Academy of Chinese Medical Sciences (Approval No. 2024-214-KY), the Medical Ethics Committee of Xiyuan Hospital, China Academy of Chinese Medical Sciences (Approval No. 2025XLA082-2), and the Medical Ethics Committee of Jinan Municipal Hospital of Traditional Chinese Medicine (Approval No. 2026-ky-027).

### 2.2 Diagnostic, inclusion, and exclusion criteria

The diagnosis of CHF was based on applicable guideline recommendations and required compatible symptoms and signs together with objective evidence from ancillary examinations such as echocardiography, natriuretic peptides, electrocardiography, or chest imaging (11, 12). Eligible participants were 18–85 years of age and met the diagnostic criteria for chronic heart failure.

Patients were excluded if they did not fulfill the inclusion criteria; had advanced liver disease (e.g., decompensated cirrhosis or Child–Pugh class C) or severe renal impairment (e.g., eGFR <30 mL/min/1.73 m² or dialysis requirement); had acute infection exacerbations that significantly affected NLR or TyG levels; or lacked essential clinical, laboratory, or imaging data for analysis.

### 2.3 Data collection and processing

Clinical data were extracted retrospectively through a multimodal hospital data system. All predictors used for model development were obtained at baseline and before the first documented AF event. The collected variables included: (1) demographic characteristics and vital signs, such as sex, age, temperature, heart rate, respiratory rate, and blood pressure; (2) comorbidities and medical history, including chronic kidney disease, bradycardia, arrhythmia, anxiety, depression, hypertension, diabetes, stroke, pneumonia, anemia, and other relevant conditions; (3) inflammatory and immune markers, including NLR, PLR, SII, SIRI, and lactate dehydrogenase (LDH); (4) metabolic and biochemical variables, including the TyG index, alkaline phosphatase (ALP), immunoglobulin M (IgM), hemoglobin A1c, electrolytes, complement-related variables, and fibrin(ogen) degradation products (FDP); and (5) routine hematological indices, such as white blood cell count, red blood cell count, hemoglobin, hematocrit, eosinophils, and red cell distribution width-standard deviation.

Derived indices were calculated as follows: NLR = neutrophil count/lymphocyte count; PLR = platelet count/lymphocyte count; SII = platelet count × neutrophil count/lymphocyte count; SIRI = neutrophil count × monocyte count/lymphocyte count; and TyG = ln [fasting triglycerides (mg/dL) × fasting blood glucose (mg/dL)/2].

Diagnostic definitions for comorbidities are summarized in **Supplementary Table 1**.

### 2.4 Missing data processing

To address missing data, K-nearest neighbor (KNN) imputation was applied to variables with a missing data rate of less than 20%. Variables with a missing data rate greater than 20% were excluded to reduce errors caused by missing values.

### 2.5 Definition of the outcome

In this study, recent-onset AF was defined as the outcome event, as follows:

Diagnosis of recent-onset AF: The diagnostic criteria were based on the 2014 Focused Update of the Canadian Cardiovascular Society Guidelines for the Management of Atrial Fibrillation (13). Eligible participants had no history of any form of atrial fibrillation or atrial flutter at baseline. During follow-up, AF events were defined as those first documented by standard electrocardiographic monitoring. A standard 12-lead electrocardiogram or a single-lead electrocardiographic recording of ≥30 s showing an AF rhythm, namely the absence of identifiable P waves and irregular RR intervals, was considered diagnostic.

### 2.6 Statistical analysis

All the statistical analyses were conducted via R version 4.3.3 (R Foundation for Statistical Computing, Vienna, Austria) and Zstats statistical software. Patients were categorized according to comorbidity status (0/1) and sex (male = 1, female = 2). Normality was assessed via the Kolmogorov–Smirnov test. Normally distributed continuous variables are expressed as the mean ± standard deviation (SD), and differences between two groups were assessed via the independent-samples t-test. Continuous variables that were not normally distributed are reported as medians (P25, P75), and comparisons between groups were conducted via the nonparametric rank-sum test. Categorical variables are presented as frequencies and percentages (%), and comparisons between groups were performed via Fisher’s exact test or the Pearson chi-square test.

Restricted cubic spline analysis with four knots at the 5th, 35th, 65th, and 95th percentiles was first used to assess the overall and nonlinear associations of the selected continuous variables with recent-onset AF, using the median as the reference. For variables showing potential nonlinearity, segmented logistic regression was subsequently performed using the segmented package in R (14). A conventional logistic model was compared with a two-piecewise logistic model, and The change point and segment-specific odds ratios with 95% confidence intervals were estimated. Model fit was compared using the likelihood ratio test, while the Davies test assessed changes in slope; Holm and Benjamini–Hochberg corrections were applied for multiple testing (15, 16). Bidirectional mediation analyses were performed for the TyG–NLR–AF and NLR–TyG–AF pathways using the mediation package in R. Linear and logistic regression models were fitted for the continuous mediator and binary AF outcome, respectively. Total, indirect, and direct effects and the proportion mediated were estimated using bootstrap confidence intervals. The original model was used for the primary analysis, whereas an expanded model was used as a sensitivity analysis (17–19). In addition, in both the mediator variable model and the outcome model, calculate the VIF or GVIF for the exposure variable, the mediator variable, and all covariates to assess multicollinearity (20, 21). Subgroup analyses were performed to assess the robustness of the associations of the NLR and TyG with recent-onset AF in patients with CHF. Two-sided *P* values were reported throughout, and values less than 0.05 were considered statistically significant.

### 2.7 Model development and comparison

Machine learning models were developed via Python 3.12 (https://www.python.org). During model development, the dataset was randomly divided into a training set (70%) and a test set (30%). Prediction models were constructed using the selected variables, and nine machine learning algorithms were applied to predict recent-onset AF in patients with CHF, including gradient boosting, K-nearest neighbors (KNN), decision tree, random forest, artificial neural network (ANN), support vector machine (SVM), the Light Gradient Boosting Machine (LightGBM), extra trees (ET), and extreme gradient boosting (XGBoost).

Several commonly used evaluation metrics including the area under the receiver operating characteristic curve (AUC-ROC), accuracy, sensitivity, specificity, F1 score, positive predictive value (PPV), negative predictive value (NPV), and Cohen’s kappa, have been employed to assess model reliability and performance, including key model performance measures. KNN imputation, feature selection, feature transformation, and hyperparameter tuning were performed using the training data only. Calibration performance was assessed using the Brier score, calibration-in-the-large, calibration slope, observed-to-expected event ratio, and calibration curves comparing observed event frequencies with predicted risks. A fixed random seed of 2026 was used. AUCs were compared using the DeLong test, and Decision curve analysis (DCA) was performed to assess clinical utility. The continuous net reclassification improvement (NRI) and integrated discrimination improvement (IDI) were calculated against the conventional clinical model, with 95% CIs estimated from 1, 000 bootstrap resamples. Calibration-in-the-large was estimated as the intercept of a logistic calibration model with the logit-transformed predicted probability included as an offset, whereas the calibration slope was estimated by regressing the binary outcome on the logit-transformed predicted probability. The fitted preprocessing procedures were then applied unchanged to the internal test and external validation sets (22, 23).

### 2.8 Model interpretability

SHapley Additive exPlanations (SHAP) is a technique used to interpret predictions generated by machine learning models. SHAP quantifies the contribution of each feature to the predicted probability for the outcome and allows both global interpretation (overall feature importance) and local interpretation (feature contributions for an individual patient). Larger absolute SHAP values indicate greater influence on model output; positive SHAP values increase predicted risk, whereas negative values decrease it (24).

## 3 Results

### 3.1 Patient selection

A total of 15, 031 patients with CHF were identified through the multimodal data system, including 8, 645 patients admitted to Guang’anmen Hospital between January 2015 and December 2024 and 6, 386 patients from Xiyuan Hospital. Patients were excluded if they had more than 20% missing laboratory data (n = 4, 227), were younger than 18 years or older than 85 years (n = 1, 480), had severe hepatic or renal disease (n = 1, 029), or experienced an acute infectious exacerbation that substantially influenced the NLR or TyG level (n = 851). For individuals with multiple hospital admissions, only the first hospitalization was retained (n = 2, 572). Finally, 4, 872 patients were included in the cohort analysis. Data from CHF patients at Jinan Municipal Hospital of Traditional Chinese Medicine who met the inclusion and exclusion criteria were used as the validation set (n = 277). The detailed flowchart is shown in **Supplementary Figure 1**.

### 3.2 Baseline characteristics

A total of 4, 872 patients were included in this study, of whom 3, 582 were in the control group, accounting for 73.52%, and 1, 290 had recent-onset AF, accounting for 26.48%. Significant between-group differences were found in age, HR, respiratory rate, SBP, NLR, PLR, SII, SIRI, TyG, LDH, the Baso ratio, the Eos count, the Eos ratio, WBC, Casts, HbA1c, RBC, RDW-SD, hematocrit, MCV, FDP, CYFRA21, hemoglobin, C3, C4, magnesium, chronic kidney disease, hypertension, diabetes, stroke, pneumonia, and anemia (*P* < 0.05). The details are presented in **Supplementary Table 2**.

### 3.3 Model development and comparison

#### 3.3.1 Variable screening and selection

**Figure 1:**
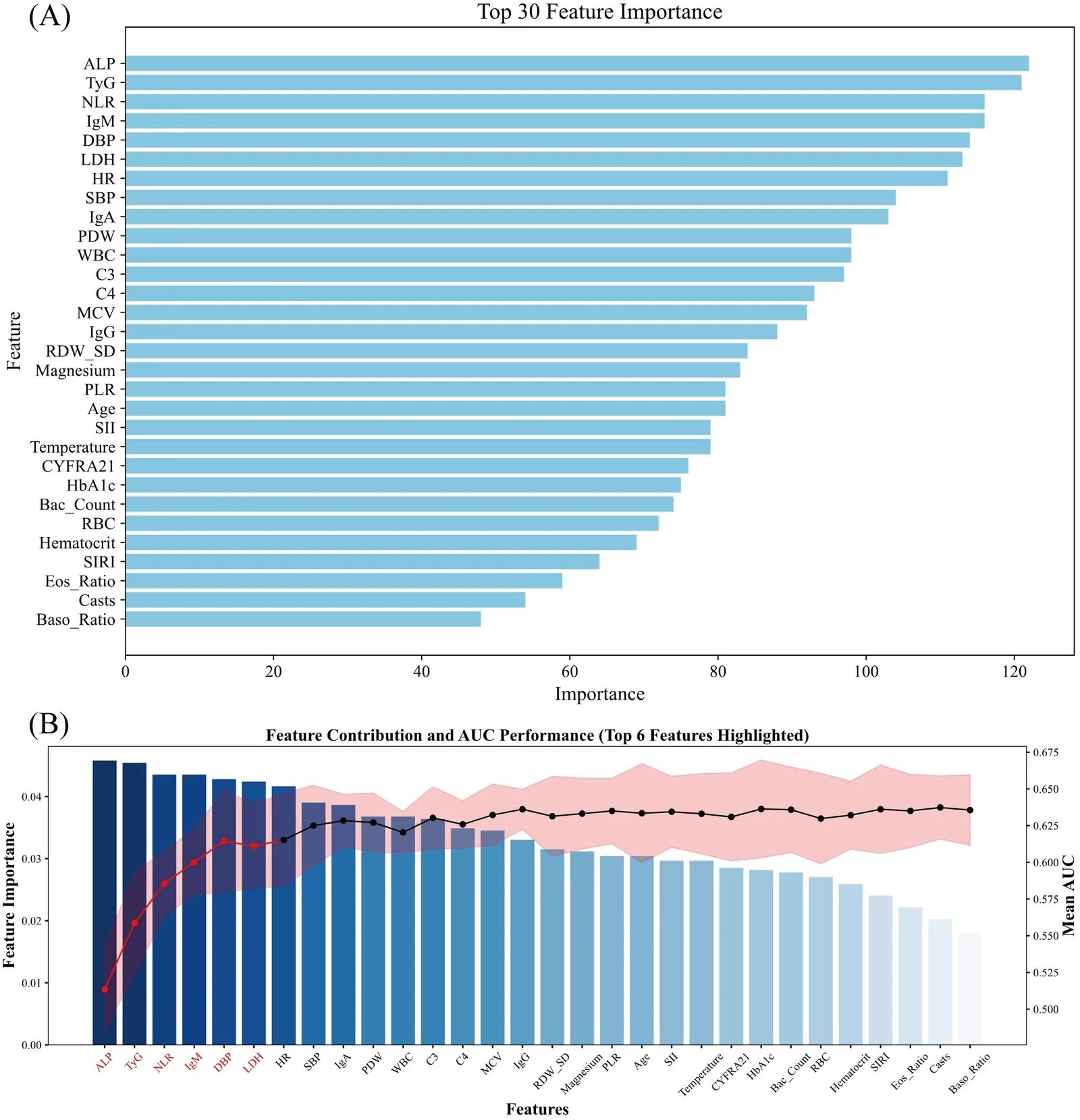
LightGBM model for variable selection. **(A)** Obtain variable importance. **(B)** The LGBM classifier evaluates the contribution of variables on the basis of the AUC.

Variable selection was comprehensively assessed using two complementary approaches. First, LightGBM model was used to rank variable importance. After training the LightGBM model and obtaining feature importance scores, all variables were ranked, and the top 30 variables were retained. These variables were then sequentially added to the model according to their importance rankings to evaluate their incremental contribution to model performance. Model discrimination was assessed using the area under the receiver operating characteristic curve (AUC). After the sixth variable was included, the addition of further variables did not materially improve the AUC; therefore, the top six variables were selected for model training and development. Second, LASSO regression was initially applied to screen all candidate variables, and the top 30 variables were subsequently ranked using the Boruta algorithm, from which the six most important variables were selected. Although the relative rankings of the six variables differed between the two approaches, the selected variable set was identical. The final selected variables were ALP, TyG, NLR, IgM, DBP, and LDH. Further details are presented in **Figure 1** and **Supplementary Figure 2**.

#### 3.3.2 Model development and hyperparameter tuning

Nine machine learning algorithms were trained to derive prediction models, including ANN, GBM, DT, KNN, LGBM, SVM, RF, extremely randomized trees, and XGB. Different hyperparameters were defined for each model, and a grid search was performed via GridSearchCV to identify the optimal parameter configuration. Each model was evaluated via 10-fold cross-validation to ensure stable performance across different data subsets.

**Figure 2:**
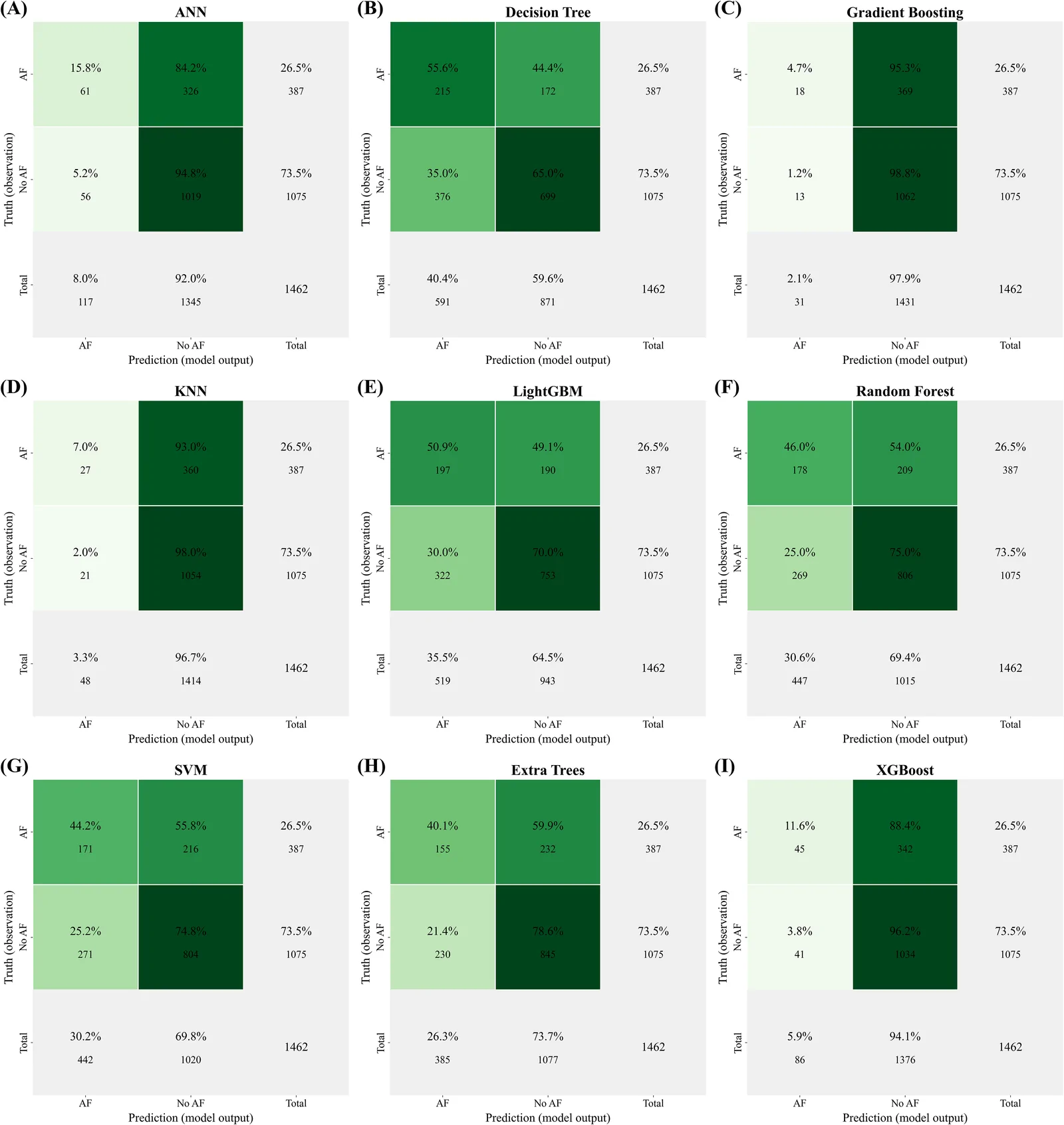
Confusion matrices of 9 machine learning models. **(A)** Confusion matrix of the ANN. **(B)** Confusion matrix of DT. **(C)** Confusion matrix of GBM. **(D)** Confusion matrix of KNN. **(E)** Confusion matrix of LightGBM. **(F)** Confusion matrix of RF. **(G)** Confusion matrix of the SVM. **(H)** Confusion matrix of ET. **(I)** Confusion matrix of XGBoost.

**Figures 2A–I** presents the confusion matrices of the nine machine learning models. The horizontal axis represents the predicted class, whereas the vertical axis represents the observed class. AF and No AF denote patients with and without recent-onset atrial fibrillation, respectively. The values in each cell represent the corresponding numbers and proportions of patients classified as true positive, false positive, false negative, and true negative. The percentages in each cell indicate the predictive accuracy of the model for that category. The numbers in each cell indicate the corresponding sample size. These matrices show the comparison between the predicted results and the actual observed results of the models.

#### 3.3.3 Model evaluation

**Figure 3:**
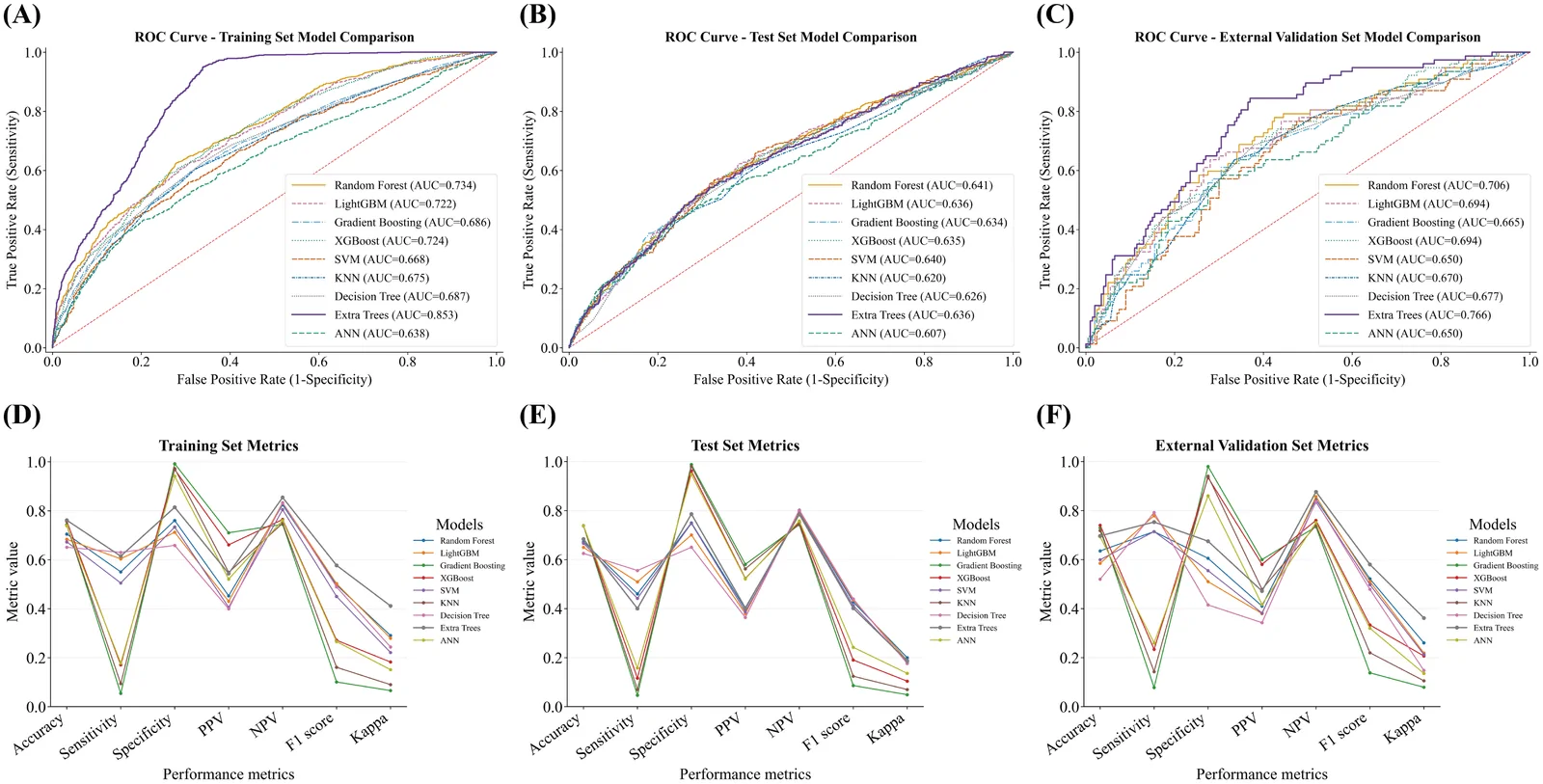
Comparison of the AUC values and classification metrics of 9 machine learning models. **(A)** AUC values of the training set. **(B)** AUC values of the test set. **(C)** AUC values of the validation set. **(D)** Classification metrics of the training set. **(E)** Classification metrics of the test set. **(F)** Classification metrics of the validation set.

**Figures 3A, B** compares the AUCs of the nine machine learning models in the training and internal test sets, while **Figures 3D, E** summarizes their classification performance, including accuracy, sensitivity, specificity, F1 score, PPV, NPV, and kappa. Among the candidate models, ET achieved the highest training AUC of 0.853 (95% CI: 0.832–0.874), with an accuracy of 0.708, sensitivity of 0.575, specificity of 0.756, and F1 score of 0.510. It also showed relatively favorable NPV and kappa values in both datasets, supporting the best overall performance.

Decision curve analysis demonstrated a positive and comparatively favorable net benefit across a broad range of low-to-moderate threshold probabilities in the training and internal test sets (**Supplementary Figures 3A, B**). Calibration was also acceptable, with Brier scores of 0.180 and 0.185, calibration intercepts of −0.002 and 0.035, calibration slopes of 1.095 and 0.919, and observed-to-expected ratios of 0.998 and 1.025, respectively (**Supplementary Figures 3D, E**; **Supplementary Tables 3**, **5**). The ET model was ultimately identified as the optimal model. The adjusted model parameters were as follows: {‘max_depth’: 15, ‘max_features’: ‘sqrt’, ‘min_samples_leaf’: 10, ‘min_samples_split’: 50, ‘n_estimators’: 300}.

#### 3.3.4 Validation

The external validation dataset consisted of a CHF cohort from Jinan Municipal Hospital of Traditional Chinese Medicine, including a total of 277 patients, among whom 200 were in the control group, accounting for 72.20%, and 77 had recent-onset AF, accounting for 27.80%. The detailed baseline characteristics are shown in **Supplementary Table 4**. This dataset was used for additional analysis to assess the consistency of model validation in an independent external dataset and its possible ability to predict recent-onset AF.

As shown in **Figures 3C, F**, ET retained the best discrimination, with an AUC of 0.766 (95% CI: 0.696–0.837), accuracy of 0.698, sensitivity of 0.753, specificity of 0.675, and F1 score of 0.580. It also performed favorably in terms of NPV and kappa. Decision curve analysis indicated a comparatively favorable net benefit across much of the clinically relevant threshold range (**Supplementary Figure 3C**). Although the external calibration curve showed greater variability, calibration remained acceptable, with a Brier score of 0.188, calibration intercept of −0.176, calibration slope of 0.936, and observed-to-expected ratio of 0.893, indicating mild overall risk overestimation but limited deviation from the ideal calibration slope (**Supplementary Figure 3F**; **Supplementary Table 5**).

#### 3.3.5 SHAP interpretability analysis

**Figure 4:**
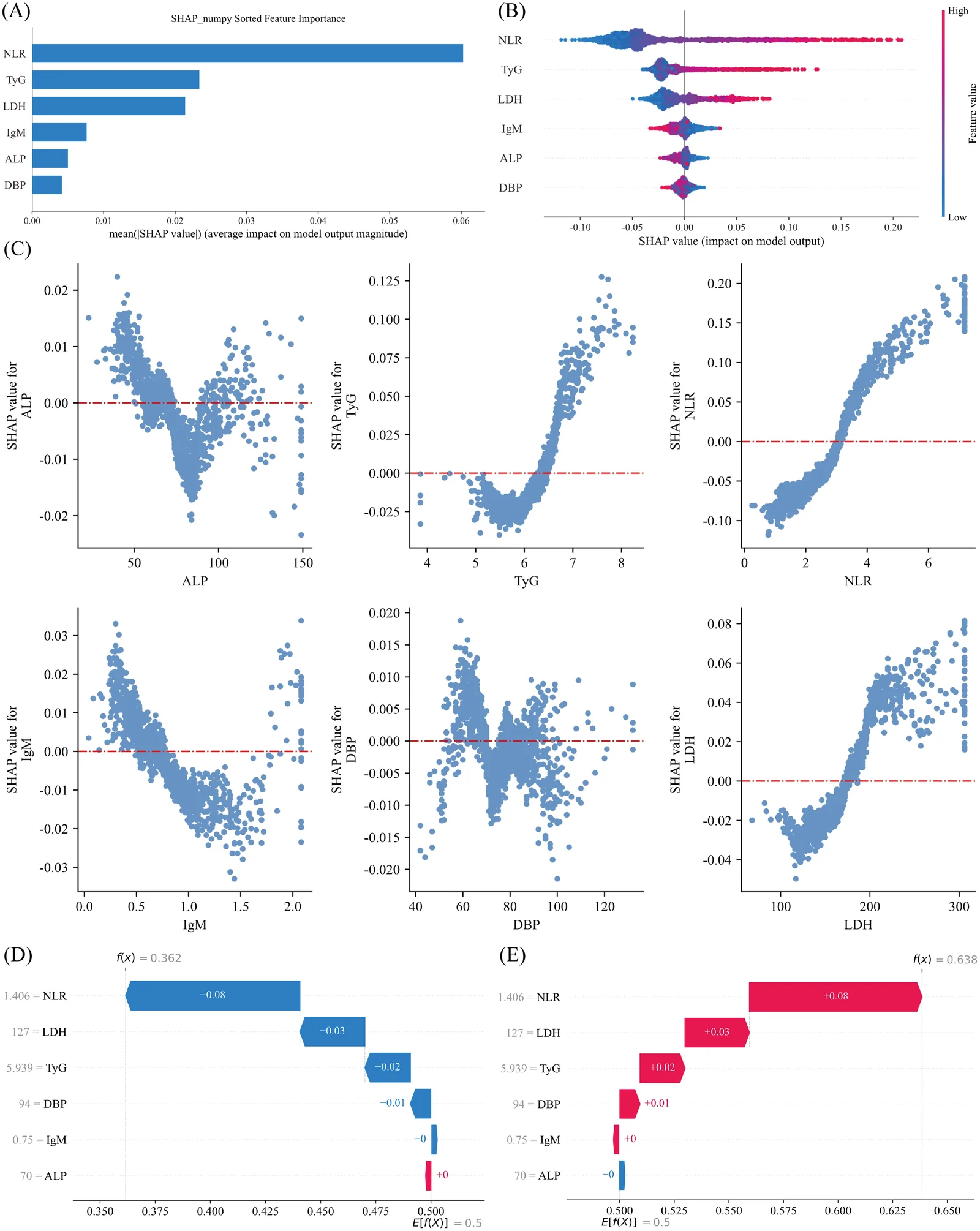
Global model interpretation via the SHAP method. **(A)** Bar chart of variable contributions. **(B)** Beeswarm plot. **(C)** Scatter plot. **(D)** Waterfall plot for CHF patients without AF. **(E)** Waterfall plot for CHF patients with recent-onset AF.

**Figure 4A** presents a SHAP bar plot used to quantify and visualize the contribution of each variable to model prediction. The variables ranked in descending order of contribution were NLR, TyG, LDH, IgM, ALP, and DBP. The results showed that the NLR was the core indicator for predicting recent-onset AF in patients with CHF. TyG made a substantial contribution to the predictive ability of the model and ranked second, whereas LDH ranked third in contribution to model prediction. IgM, ALP, and DBP contributed relatively less to the predictive performance of the model and had weaker effects.

**Figure 4B** shows a beeswarm plot of SHAP values for individual features, illustrating how each feature influenced the model prediction over a range of values. Among all the features, the NLR and TyG index had the greatest impact on the SHAP values, and higher values (shown in purple) corresponded to higher SHAP values, indicating that higher NLR and TyG index values had a stronger effect on model prediction. LDH was also an important feature, with a wide distribution of SHAP values, and higher LDH values increased the model output. Features such as IgM, ALP, and DBP contributed less to model prediction. These results indicate that the model relied mainly on NLR- and TyG-related indicators, whereas factors such as IgM, ALP, and DBP had less influence.

**Figure 4C** shows SHAP value scatter plots for each feature, illustrating how individual features influence the model output across different value ranges. As shown in the figure, as the values of NLR, TyG, and LDH increased, they exerted a significant positive influence on the model’s predicted risk, indicating that elevated levels were generally associated with a greater likelihood of recent-onset AF. In contrast, IgM, ALP, and DBP showed an approximately inverse relationship.

**Figures 4D, E** presents waterfall plots of SHAP values for an individual patient, demonstrating how different features contributed to the model prediction. Each bar represents a single feature, with blue indicating a negative effect on the predicted outcome (risk reduction) and red indicating a positive effect (risk increase). According to the waterfall plots, the NLR, TyG, and LDH were the main influencing factors for recent-onset AF in patients with CHF, and as the NLR, TyG, and LDH increased, the risk of recent-onset AF also increased accordingly.

#### 3.3.6 Feature combination and predictive performance analysis

**Figure 5:**
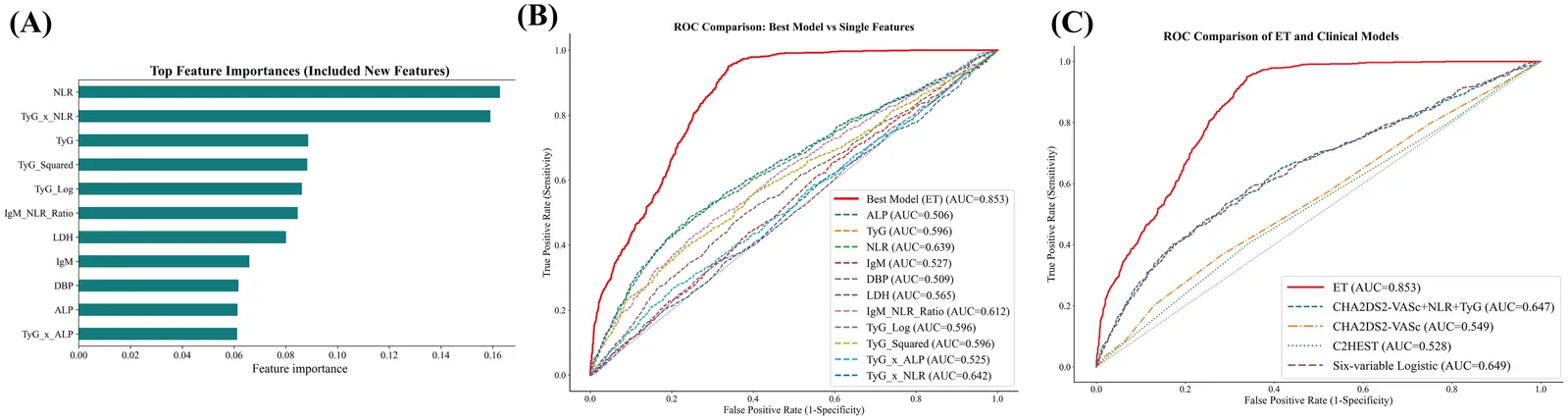
Feature importance and predictive performance of the ET model, individual indicators, and clinical models. **(A)** Feature importance of the original and derived variables in the ET model. **(B)** ROC curves comparing the ET model with individual variables and derived indicators. **(C)** ROC curves comparing the ET model with CHA_2_DS_2_-VASc+NLR+TyG, CHA_2_DS_2_-VASc, C_2_HEST, and the six-variable logistic regression model. CHA_2_DS_2_-VASc assigns 1 point each for CHF/LV dysfunction, hypertension, age 65–74 years, diabetes, vascular disease, and female sex, and 2 points each for age ≥75 years and prior stroke/TIA/systemic embolism (25). C_2_HEST assigns 1 point each for coronary artery disease, COPD, hypertension, and hyperthyroidism, and 2 points each for age ≥75 years and systolic heart failure (26).

To further explore the synergistic predictive value among indicators, combination analyses were performed for the six variables, namely NLR, TyG, LDH, IgM, ALP, and DBP. The results revealed that TyG×NLR and NLR alone had the greatest feature importance and the best predictive performance among the individual and combined indicators, with ROC-AUC values of 0.642 and 0.639, respectively. Compared with conventional clinical models, including CHA_2_DS_2_-VASc and C_2_HEST, the ET model retained superior discrimination. Details are shown in **Figures 5A–C**.

### 3.4 Threshold effect analysis of TyG and the NLR

**Figure 6:**
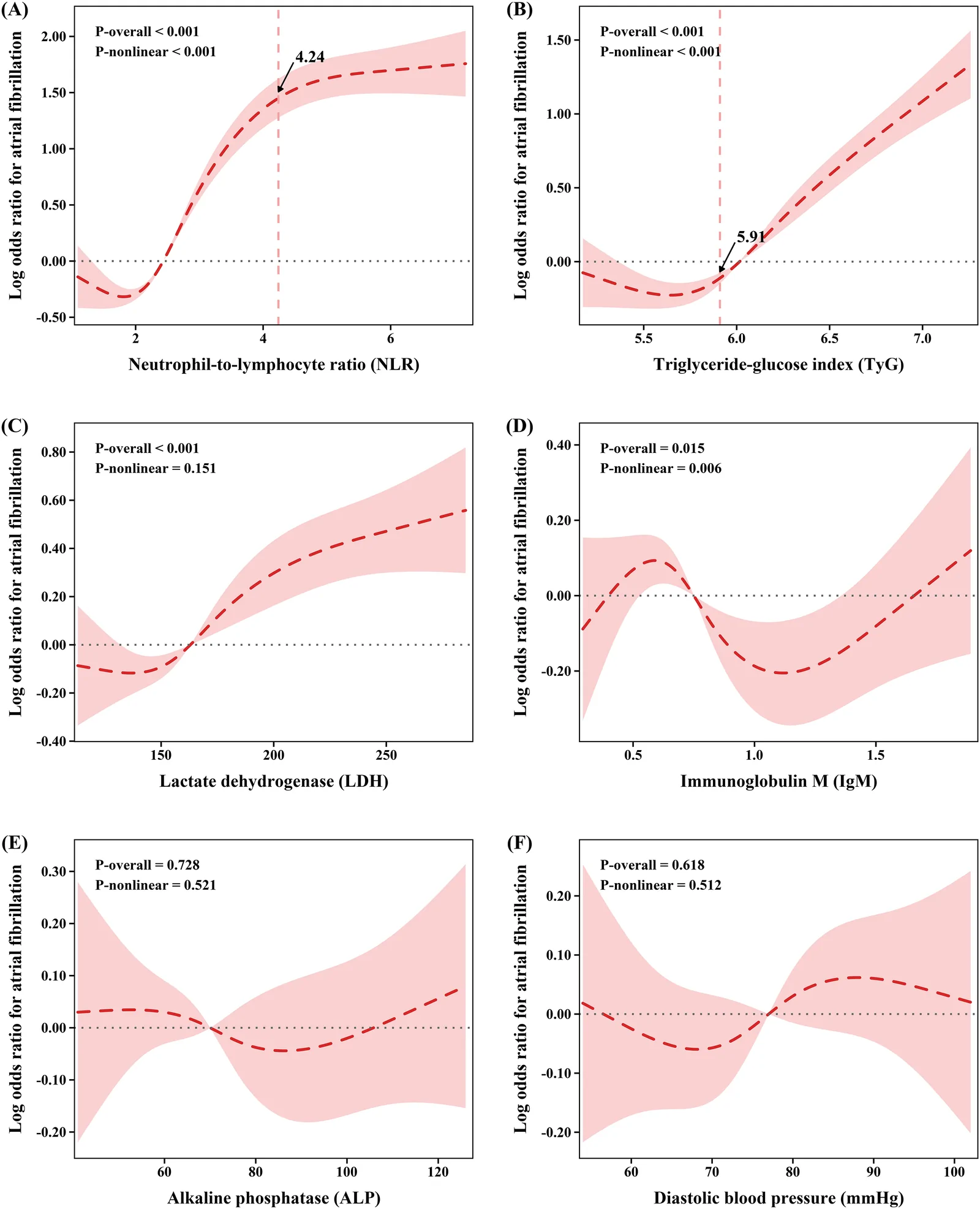
Threshold effect analysis of each indicator. **(A)** threshold effect analysis of the NLR; **(B)** threshold effect analysis of TyG; **(C)** threshold effect analysis of LDH; **(D)** threshold effect analysis of IgM; **(E)** threshold effect analysis of ALP; **(F)** threshold effect analysis of DBP.

SHAP and restricted cubic spline analyses indicated significant overall and nonlinear associations of both NLR and TyG with recent-onset AF (all *P* for overall and nonlinearity <0.001; **Figures 6A, B**). Segmented regression identified change points at 4.24 for NLR and 5.91 for TyG. Below the NLR change point, the association with AF was pronounced (OR, 1.97; 95% CI, 1.79–2.17; *P* < 0.001), whereas it was substantially attenuated above 4.24 (OR, 1.21; 95% CI, 1.04–1.41; segment *P* = 0.013). The segmented model showed better fit than the linear model (AIC, 5378.819 vs 5413.184), and the change point remained significant after Holm and Benjamini–Hochberg corrections. Details are shown in **Figure 6A** and **Supplementary Table 6**.

For TyG, no significant association was observed below 5.91 (OR, 0.91; 95% CI, 0.59–1.40; *P* = 0.667), whereas TyG was strongly associated with AF above this threshold (OR, 3.01; 95% CI, 2.49–3.62; *P* < 0.001). The segmented model also improved model fit (AIC, 5607.606 vs 5630.846), with the change point remaining significant after Holm and Benjamini–Hochberg corrections. Details are shown in **Figure 6B** and **Supplementary Table 6**. LDH was associated with AF without evidence of nonlinearity (*P* for overall <0.001; *P* for nonlinearity=0.151), whereas IgM showed significant overall and nonlinear associations (*P* = 0.015 and 0.006, respectively). No significant overall or nonlinear associations were observed for ALP or DBP (**Figures 6C–F**).

### 3.5 Subgroup analysis of NLR and TyG levels and the risk of recent-onset AF in patients with CHF

**Figure 7:**
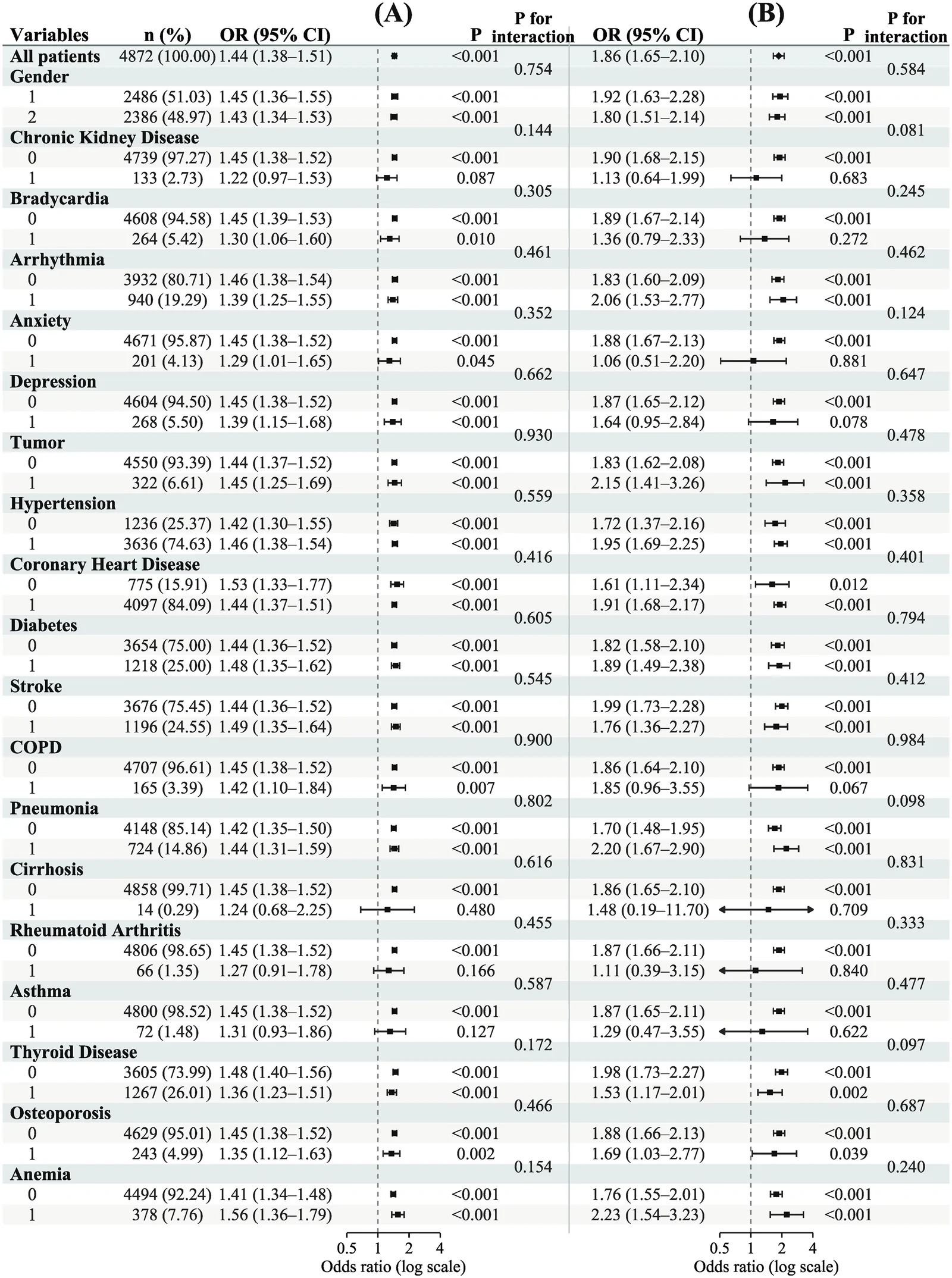
Subgroup analysis of recent-onset atrial fibrillation occurrence in CHF patients with different NLR and TyG levels. **(A)** NLR levels.; **(B)** TyG levels.

To further evaluate the robustness of the associations between the NLR and TyG level and the risk of recent-onset AF, subgroup analyses were performed in this study, with the occurrence of recent-onset AF as the dependent variable (coded as: no = 0, yes = 1) and the NLR and TyG level as the independent variables. The results revealed that the NLR was significantly associated with the risk of recent-onset AF across subgroups stratified by sex, bradycardia, arrhythmia, anxiety, depression, tumor, hypertension, coronary heart disease, diabetes, stroke, COPD, pneumonia, thyroid disease, osteoporosis, and anemia (*P* < 0.05). TyG was significantly associated with the risk of recent-onset AF across subgroups stratified by sex, arrhythmia, tumor, hypertension, coronary heart disease, diabetes, stroke, pneumonia, thyroid disease, osteoporosis, and anemia (*P* < 0.05). Effect estimates were generally positive across subgroups. No statistically significant interactions were detected. (*P* for interaction > 0.05) (see **Figures 7A, B**).

### 3.6 Mediation analysis of the NLR, TyG, and recent-onset AF

**Figure 8:**
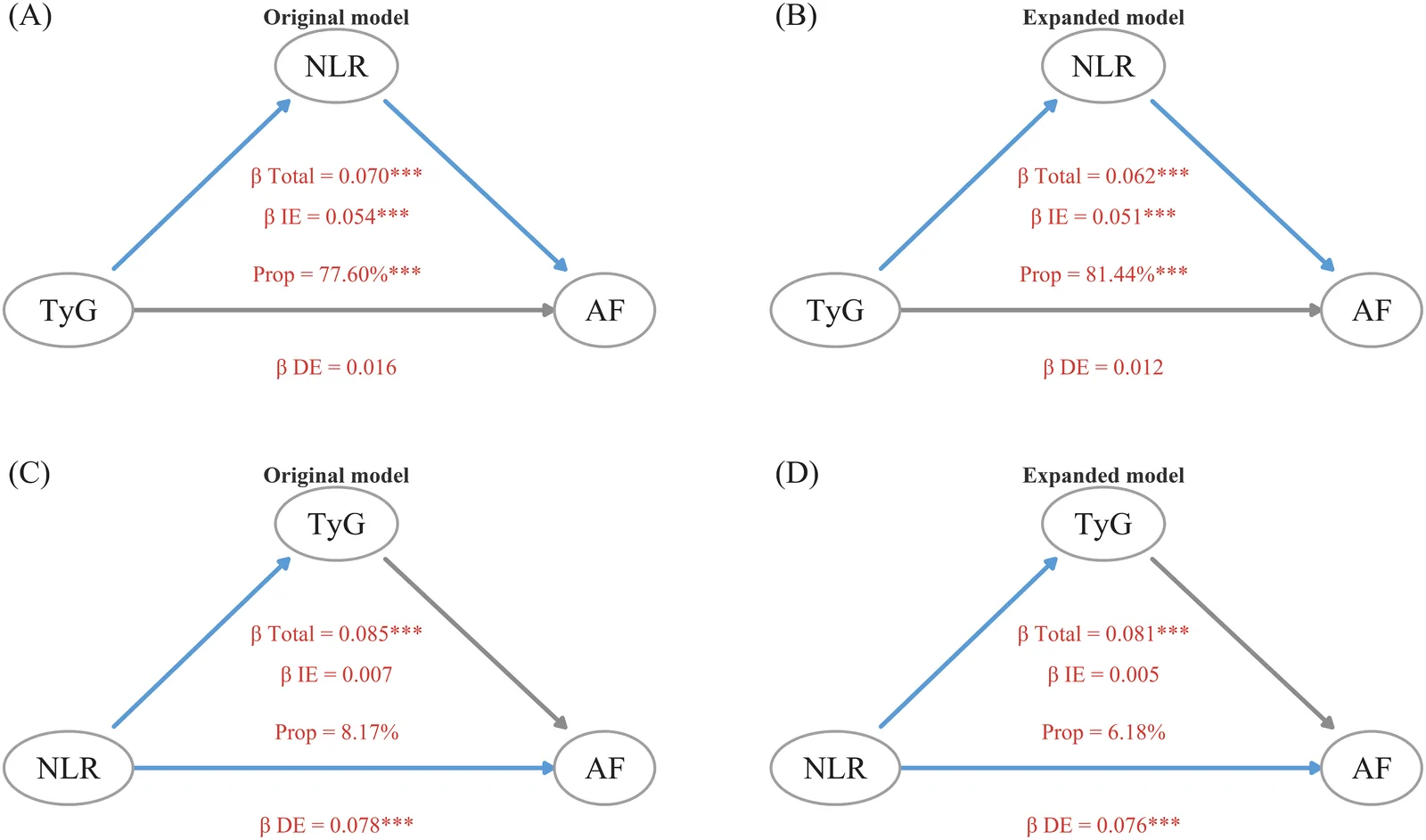
Bidirectional mediation analysis of TyG, NLR, and recent-onset atrial fibrillation. **(A)** TyG–NLR–AF pathway in the original model; **(B)** TyG–NLR–AF pathway in the expanded model; **(C)** NLR–TyG–AF pathway in the original model; **(D)** NLR–TyG–AF pathway in the expanded model. The original model was adjusted for sex, CKD, bradycardia, arrhythmia, anxiety, depression, tumor, hypertension, CHD, diabetes, stroke, COPD, pneumonia, cirrhosis, rheumatoid arthritis, asthma, thyroid disease, osteoporosis, and anemia. As a sensitivity analysis, the expanded model additionally incorporated age, temperature, heart rate, respiratory rate, systolic blood pressure, diastolic blood pressure, lactate dehydrogenase, haemoglobin, fibrin degradation products, and magnesium. β Total, β IE, and β DE represent the total, indirect, and direct effects, respectively; Prop represents the proportion mediated. *P < 0.05, **P < 0.01, ***P < 0.001. NLR, neutrophil–lymphocyte ratio; TyG, triglyceride–glucose index; AF, recent-onset atrial fibrillation.

To explore the statistical relationships among TyG, NLR, and recent-onset AF under two prespecified model specifications, mediation analyses were performed using the original model, while an expanded model with additional covariate adjustment was used as a sensitivity analysis to assess the robustness of the findings. In the original model, when the NLR was analyzed as the exposure variable in the NLR–TyG–AF pathway, the proportion mediated by TyG was low and not statistically significant (*prop* = 8.17%). The direct effect remained significant (β = 0.078, *P* < 0.001), whereas the indirect effect through TyG was small (β = 0.007), indicating that the association between the NLR and recent-onset AF was primarily attributable to the direct pathway. Consistent results were observed in the expanded model, with a mediated proportion of 6.18%, a significant direct effect of 0.076, and a small indirect effect of 0.005, supporting the robustness of the primary findings (**Figures 8C, D**).

When TyG was analyzed as the exposure variable in the TyG–NLR–AF pathway, the estimated indirect association through NLR accounted for a substantial proportion of the TyG–AF association and was statistically significant in the original model (*prop* = 77.60%, *P* < 0.001). The total effect was 0.070, of which the indirect effect through the NLR accounted for 0.054, whereas the remaining direct effect was relatively small (β = 0.016). In the expanded model, the mediated proportion remained substantial (prop = 81.44%, *P* < 0.001), with a total effect of 0.062, an indirect effect of 0.051, and a direct effect of 0.012 (**Figures 8A, B**). The broadly consistent effect estimates and overlapping confidence intervals between the original and expanded models further supported the stability of the mediation findings. In addition, the adjusted generalized variance inflation factors were close to 1, indicating no substantial multicollinearity in either mediation model. The detailed confidence intervals are shown in **Supplementary Figures 4** and **5**, and the collinearity diagnostics are provided in **Supplementary Table 7**.

## 4 Discussion

In this multicenter retrospective study, we developed and externally validated an interpretable ML framework for predicting recent-onset AF in patients with CHF. Among nine candidate algorithms, the ET model was selected as the final model based on the overall balance of discrimination, calibration, classification performance, and clinical net benefit. The model relied on a compact six-feature panel composed of NLR, TyG, LDH, IgM, ALP, and DBP, among which NLR and TyG were the most influential predictors. Additional threshold, subgroup, and exploratory mediation analyses consistently highlighted the predictive relevance of concurrent metabolic and inflammatory abnormalities in patients with CHF and recent-onset AF.

These findings are biologically and clinically plausible, as CHF and AF are closely interconnected conditions that share multiple mechanisms, including hemodynamic overload, atrial stretch, fibrosis, autonomic imbalance, oxidative stress, inflammation, and impaired myocardial energy metabolism (27, 28). In this context, biomarkers reflecting inflammatory and metabolic stress may provide a more integrated signal of arrhythmogenic vulnerability than isolated conventional variables (29, 30). In our study, NLR emerged as the most important predictor in the ET model. As an inexpensive, reproducible, and readily available index derived from routine blood counts, NLR reflects enhanced neutrophil-driven innate immune activation together with relative lymphocyte reduction, a pattern associated with oxidative injury, endothelial dysfunction, cytokine release, fibrosis-related signaling, and electrical remodeling, all of which may promote atrial arrhythmogenesis (31, 32). Beyond innate inflammatory responses, T-cell dysfunction may represent another important mechanistic link between abnormalities in glucose–lipid metabolism and AF. Metabolic stress can reprogram T-cell function by enhancing proinflammatory CD4^+^ and CD8^+^ T-cell responses while disrupting regulatory T-cell-mediated immune homeostasis within ischemic and inflammatory environments. Activated T cells may promote myocardial inflammation, oxidative stress, and fibroblast activation through the secretion of cytokines such as interferon-γ (IFN-γ), tumor necrosis factor-α (TNF-α), and interleukin-17 (IL-17), thereby contributing to atrial electrical and structural remodeling (33). In addition, tissue-resident memory T cells within epicardial adipose tissue may alter calcium handling in atrial cardiomyocytes and activate inflammation- and apoptosis-related signaling pathways, whereas senescent CD8^+^ T cells may participate in AF development by increasing IFN-γ release and inducing abnormalities in calcium handling (34). Notably, its association with recent-onset AF was nonlinear, with risk rising steeply below 4.24 and remaining positive but attenuated above this threshold, suggesting a possible threshold or saturation effect.

TyG, the second most important predictor, also showed a significant threshold effect and was positively associated with recent-onset AF mainly above 5.91. As a surrogate marker of insulin resistance, TyG captures the combined burden of dysglycemia and hypertriglyceridemia and has been linked to adverse cardiovascular outcomes in previous studies (35, 36). Mechanistically, insulin resistance may promote free fatty acid overload, oxidative stress, mitochondrial dysfunction, endothelial injury, and chronic low-grade inflammation, thereby accelerating atrial structural and electrical remodeling (37, 38). Previous studies, mainly in patients with type 2 diabetes, reported an approximately linear positive association between TyG and AF risk (39). This difference may reflect the unique metabolic characteristics of CHF, in which lower TyG levels may also indicate malnutrition, cardiac cachexia, or reduced metabolic reserve rather than favorable metabolic status (40).

Together, these findings support the importance of the metabolic-inflammatory axis in AF development among CHF patients. Although the full ET model outperformed any single marker or simplified combination, the relatively strong performance of NLR alone and the TyG × NLR combination further suggests that inflammation and metabolic dysfunction may act synergistically rather than independently in shaping arrhythmic risk, consistent with observations in other cardiovascular settings (41).

One of the most interesting findings of this study is the asymmetric mediation pattern between TyG and NLR. When TyG was treated as the exposure, a large proportion of its association with AF was mediated through NLR, whereas TyG explained only a small fraction of the NLR-AF association when the direction was reversed (42). This pattern suggests that inflammation may act as a major downstream effector pathway linking metabolic disturbance to AF development. Such a model is consistent with the concept of immunometabolism, whereby insulin resistance and lipid overload activate innate immune pathways, inflammasome signaling, cytokine release, and myeloid-cell reprogramming, ultimately promoting tissue remodeling and electrical instability (43, 44). In contrast, the direct effect of NLR on AF remained dominant, implying that once systemic inflammation is established, it may contribute to AF risk independently of TyG. Although causal inferences from retrospective observational data must be made cautiously, these findings provide a hypothesis-generating statistical framework that warrants further mechanistic and prospective investigation (29). These observations may also have therapeutic implications.

Our findings suggest that controlling glucose or lipid levels alone may be insufficient in metabolically high-risk patients once inflammatory activation has already been established (45). In particular, patients with concomitant dysglycemia, lipid metabolic disturbance, and heightened inflammatory burden may warrant greater clinical attention, as they may represent a subgroup at especially high risk of AF and may benefit from earlier and more intensive anti-inflammatory management to reduce arrhythmic occurrence (46, 47). Prior studies have shown that therapies with both metabolic and anti-inflammatory benefits, such as sodium-glucose cotransporter 2 inhibitors, can improve cardiovascular outcomes, while direct anti-inflammatory strategies, such as colchicine, may also reduce the risk of inflammation-related cardiovascular events (48–50). Although our study was not designed to evaluate treatment effects, it supports the hypothesis that prevention of recent-onset AF in CHF may require a more integrated strategy targeting both metabolic dysfunction and downstream inflammation. This possibility deserves further investigation in prospective cohorts and interventional trials. Because TyG and NLR were measured at baseline in a retrospective observational study, their temporal ordering could not be determined, and residual or unmeasured confounding cannot be excluded. These findings should therefore be interpreted as hypothesis-generating and require prospective validation using serial biomarker measurements.

In addition to NLR and TyG, the final ET model incorporated LDH, IgM, ALP, and DBP. LDH likely reflects systemic metabolic stress and tissue injury, which are relevant to CHF progression and atrial vulnerability. IgM, ALP, and DBP contributed to model discrimination as routine clinical variables that may capture immune, metabolic, hepatic, nutritional, and hemodynamic information not fully reflected by NLR and TyG alone. Their inclusion improved overall prediction, even though their individual effects were weaker and less central than those of NLR and TyG. This highlights an important feature of ML models: clinically useful prediction often arises from the joint contribution of several modest signals rather than from one dominant biomarker alone.

From a modeling perspective, ET outperformed the other tested algorithms, likely because its tree-based ensemble architecture introduces additional randomness in selecting splitting variables and thresholds, thereby reducing variance and improving generalization in complex clinical tabular data (51, 52). This advantage is particularly relevant when predictor–outcome relationships are nonlinear and involve interactions or threshold effects, as observed for NLR and TyG in this study. Unlike conventional models that often require prespecified interaction terms or nonlinear transformations, ET can capture such patterns adaptively, and its stable performance in the external validation cohort further supports its suitability for this application. Importantly, this model also has clear translational value: all six predictors are routinely available or easily derived in standard clinical practice, supporting feasibility in real-world and resource-limited settings; SHAP-based interpretation enhances transparency by showing why individual patients are classified as high risk; and the combined elevation of TyG and NLR may identify a particularly vulnerable phenotype characterized by coexisting metabolic stress and inflammation, who may benefit from closer rhythm surveillance and more intensive management of modifiable risk factors.

Overall, our work contributes in three ways. First, it provides an externally validated and interpretable ML model for AF risk prediction in CHF based on readily accessible variables. Second, it identifies NLR and TyG as the dominant predictive features and quantifies clinically relevant thresholds for both. Third, it proposes a biologically coherent framework in which metabolic disturbance may promote AF partly through inflammatory activation. Together, these findings may help bridge statistical prediction and mechanism-oriented risk stratification in CHF.

## 5 Limitations

Several limitations should be acknowledged. First, this was a retrospective multicenter study, and despite the inclusion of multiple hospitals, selection bias and information bias cannot be fully excluded. Second, the participating centers were concentrated mainly in North China, which may limit geographic generalizability. Third, the external validation cohort was relatively small (n = 277), and broader validation in larger and more diverse populations is still needed. Fourth, the model was constructed from baseline data and did not incorporate longitudinal changes in biomarkers or treatments during follow-up. Fifth, residual confounding remains possible in the threshold and mediation analyses, particularly with respect to medication exposure, disease severity, and other unmeasured factors. Finally, although NLR and TyG are practical markers, they cannot fully characterize the full spectrum of inflammation, insulin resistance, and atrial remodeling. Future prospective studies combining serial biomarker measurement, mechanistic experiments, and multi-omics profiling are needed to refine and validate the proposed metabolism-inflammation-AF pathway. In addition, TyG and NLR were measured at the same baseline time point, precluding confirmation of their temporal ordering, and the assumptions required for causal mediation could not be fully verified.

## 6 Conclusions

We developed and externally validated an interpretable ML model for predicting recent-onset AF in patients with CHF. Among nine candidate algorithms, the ET model showed the best overall performance. NLR and TyG were the most influential predictors, and both exhibited clinically relevant threshold effects. Mediation analysis further suggested that the AF risk associated with TyG may be conveyed in large part through inflammatory activation captured by NLR. These findings support the use of routine inflammatory and metabolic biomarkers for risk stratification and provide a practical framework for the early identification of CHF patients at high risk of recent-onset AF.

## Data Availability

The raw data supporting the conclusions of this article will be made available by the authors, without undue reservation.

## Glossary

ACME: average causal mediation effect
ADE: average direct effect
AF: atrial fibrillation
AIC: Akaike information criterion
ALP: alkaline phosphatase
ANN: artificial neural network
AUC: area under the receiver operating characteristic curve
BH: Benjamini–Hochberg
C₂HEST: clinical AF risk score incorporating coronary artery disease/chronic obstructive pulmonary disease, hypertension, elderly age, systolic heart failure, and thyroid disease
C3: complement component 3
C4: complement component 4
CHA₂DS₂-VASc: clinical AF risk score incorporating congestive heart failure, hypertension, age, diabetes, stroke/transient ischemic attack, vascular disease, and sex category
CHD: coronary heart disease
CHF: chronic heart failure
CI: confidence interval
CKD: chronic kidney disease
COPD: chronic obstructive pulmonary disease
DBP: diastolic blood pressure
DE: direct effect
Df: degrees of freedom
DT: decision tree
eGFR: estimated glomerular filtration rate
ET: extremely randomized trees (Extra Trees)
FDP: fibrin/fibrinogen degradation products
GBM: gradient boosting machine
GVIF: generalized variance inflation factor
HbA1c: glycated hemoglobin
HF: heart failure
HR: heart rate
IDI: integrated discrimination improvement
IE: indirect effect
IFN-γ: interferon-γ
IgA: immunoglobulin A
IgG: immunoglobulin G
IgM: immunoglobulin M
IL-17: interleukin-17
KNN: k-nearest neighbors
LASSO: least absolute shrinkage and selection operator
LDH: lactate dehydrogenase
LightGBM: Light Gradient Boosting Machine
LV: left ventricular
MCV: mean corpuscular volume
ML: machine learning
NLR: neutrophil-to-lymphocyte ratio
NPV: negative predictive value
NRI: net reclassification improvement
O/E: observed-to-expected
OR: odds ratio
PDW: platelet distribution width
PLR: platelet-to-lymphocyte ratio
PPV: positive predictive value
RBC: red blood cell count
RDW-SD: red cell distribution width-standard deviation
RF: random forest
ROC: receiver operating characteristic
SBP: systolic blood pressure
SD: standard deviation
SHAP: SHapley Additive exPlanations
SII: systemic immune-inflammation index
SIRI: systemic inflammation response index
SVM: support vector machine
TIA: transient ischemic attack
TNF-α: tumor necrosis factor-α
TyG: triglyceride-glucose index
VIF: variance inflation factor
WBC: white blood cell count
XGBoost: extreme gradient boosting
