# Supplementary material for: The metabolic-inflammatory axis in chronic heart failure: integrating the NLR and TyG index for recent-onset atrial fibrillation prediction via machine learning and mediation analysis
Source: Front Endocrinol (Lausanne). 2026 Aug 26;17:1831904. doi: 10.3389/fendo.2026.1831904 (PMC13508824; doi:10.3389/fendo.2026.1831904)
Supplement: Supplementary file 1 [file Supplementaryfile1.docx]

**The Metabolic-Inflammatory Axis in Chronic Heart Failure: Integrating the NLR and TyG Index for Recent-onset Atrial Fibrillation Prediction via Machine Learning and Mediation Analysis**

**Supplementary materials**

# Contents

[**Supplementary Tables	2**](#_Toc235303876)

[**eTable 1 Disease diagnosis description	2**](#_Toc235303877)

[**eTable 2 Baseline characteristics	5**](#_Toc235303878)

[**eTable 3 Classification metrics of nine machine-learning models in the training, internal test, and external validation set	7**](#_Toc235303879)

[**eTable 4 Validation set baseline features	8**](#_Toc235303880)

[**eTable 5 Discrimination, calibration, and reclassification metrics of nine machine-learning models in the training, internal test, and external validation set	10**](#_Toc235303881)

[**eTable 6 Segmented Regression–Derived Change Points and Multiple-Testing–Adjusted Davies Tests for NLR and TyG in Relation to Recent-Onset Atrial Fibrillation	11**](#_Toc235303882)

[**eTable 7 Variance inflation factor and generalized variance inflation factor diagnostics for the mediation models	12**](#_Toc235303883)

[**Supplementary Figures	19**](#_Toc235303884)

[**eFig 1 Patient screening flowchart	19**](#_Toc235303885)

[**eFig 2 Feature selection using least absolute shrinkage and selection operator (LASSO) regression and the Boruta algorithm	20**](#_Toc235303886)

[**eFig 3 Comparison of the decision curve analysis and calibration performance of 9 machine learning models	21**](#_Toc235303887)

[**eFig 4 Exploratory mediation analysis of the bidirectional associations among NLR, TyG, and recent-onset atrial fibrillation	22**](#_Toc235303888)

[**eFig 5 Estimated proportions mediated in the bidirectional exploratory mediation analyses of NLR, TyG, and recent-onset atrial fibrillation	22**](#_Toc235303889)

# Supplementary Tables

## eTable 1 Disease diagnosis description

| **diagnosis** | **Guidelines** | **Diagnostic Content** |
| --- | --- | --- |
| **depression** | “Recommendations on screening for depression in adults” ^1^ | Primarily characterized by a low mood, and accompanied by at least four of the following symptoms: (1) loss of interest and pleasure; (2) decreased energy or fatigue; (3) psychomotor retardation or agitation; (4) excessive self-criticism, self-blame, or guilt; (5) difficulty in thinking or a perceived decline in cognitive ability; (6) recurrent thoughts of death or suicide, or self-harming behaviors; (7) sleep disturbances, decreased appetite, or significant weight loss; (8) decreased sexual desire. |
| **peptic ulcer** | “Diagnosis and treatment of perforated or bleeding peptic ulcers: 2013 WSES position paper”^2^ | Pain is often described as burning, gnawing, or hunger-like sensation. The course of the disease is chronic and recurrent. Diagnosis is suggested by medical history and confirmed by endoscopy. |
| **symptomatic bradycardia** | “2013 ESC Guidelines on cardiac pacing and cardiac resynchronization therapy”^3^ | While experiencing sinus rhythm with a heart rate of less than 50 beats per minute (bpm), patients also exhibit clinical symptoms directly related to it, such as amaurosis syncope, dizziness, fatigue, decreased exercise tolerance, confusion, heart failure, etc. |
| **ventricular arrhythmia** | “EHRA/HRS/APHRS expert consensus on ventricular arrhythmias”^4^ | Including ventricular premature beats, ventricular tachycardia, ventricular flutter, and ventricular fibrillation. Symptoms include palpitations or symptoms related to hemodynamic compromise (dyspnea, chest discomfort, presyncope, syncope, cardiac arrest). Diagnosis is based on medical history, physical examination, and electrocardiogram. |
| **hypertension** | “2013 ESH/ESC guidelines for the management of arterial hypertension”^5^ | Without the use of antihypertensive medications, the office blood pressure measured three times on different days is ≥ 140/90 mmHg. |
| **coronary heart disease** | “AHA/ACCF Secondary Prevention and Risk Reduction Therapy: 2011 Update”^6^ | Previous or during hospitalization, coronary CT/angiography results indicated the presence of at least one main coronary artery stenosis ≥ 50%. |
| **diabetes** | “Standards of Medical Care in Diabetes 2015”^7^ | Fasting blood glucose ≥ 7.0 mmol/L, or 2-hour blood glucose after oral glucose tolerance test/random blood glucose ≥ 11.1 mmol/L. Fasting refers to at least 8 hours of no calorie intake. |
| **stroke** | “Guidelines for the primary prevention of stroke: a statement for healthcare professionals from the American Heart Association/American Stroke Association”^8^ | It manifests as sudden numbness or weakness (especially on one side), language or comprehension impairment, vision problems, headaches, dizziness, etc. A brain CT/MRI is recommended for a definitive diagnosis. |
| **pneumonia** | “Diagnosis and management of community and hospital acquired pneumonia (NICE)”^9^ | Clinical symptoms include fever, cough, and difficulty breathing. Diagnosis is made through a combination of medical history, imaging examination, assessment of disease severity, and identification of pathogenic microorganisms. |
| **chronic kidney disease** | “KDIGO Clinical Practice Guideline for Lipid Management in CKD”^10^ | Chronic renal structural and functional disorders (with a history of >3 months), including pathological damage with abnormal glomerular filtration rate (GFR), abnormalities in blood/urine components, and abnormalities in imaging examinations. |
| **liver** | “AASLD Practice Guideline: Management of adult patients with ascites due to cirrhosis 2012”^11^ | Symptoms such as fatigue, general malaise, loss of appetite, and weight loss may occur. A definitive diagnosis can be made through a combination of blood tests, imaging examinations, and liver biopsy. |
| **rheumatoid arthritis** | “2012 update of the 2008 American College of Rheumatology recommendations for the use of disease-modifying antirheumatic drugs and biologic agents in the treatment of rheumatoid arthritis”^12^ | It primarily presents with joint lesions, characterized by morning stiffness, swelling, and pain in the joints, which may progress to joint deformities. It is accompanied by anemia, elevated erythrocyte sedimentation rate (ESR), and increased C-reactive protein (CRP). |
| **asthma** | “Japanese Guideline for Adult Asthma 2014”^13^ | Clinical symptoms include wheezing, shortness of breath, chest tightness, or coughing. There is an excessive change in lung function accompanied by airflow limitation, and the bronchodilation test (BDT) is positive. |
| **thyroid dysfunction** | “Practice guidelines for hypothyroidism in adults: clinical issues and the controversy about combination therapy”^14^ | Persistent fear of heat/cold, unexplained weight changes, neck enlargement, exophthalmos, severe fatigue, or abnormal mood. Diagnosis is based on TSH, TT4, FT4, TT3, and FT3 levels. |
| **osteoporosis** | “Clinician’s Guide to Prevention and Treatment of Osteoporosis”^15^ | It manifests as lower back pain or generalized bone pain (aggravated at night or with weight bearing), which may be accompanied by muscle spasms and limited mobility. X-ray reveals sparse trabecular bone. |
| **anaemia** | “2011 BSG Guidelines for the Management of Iron Deficiency Anaemia”^16^ | Diagnosis is based on complete blood count (CBC) and examinations that reveal the volume and shape of red blood cells. |

References:

1.	Joffres M, Jaramillo A, Dickinson J, et al. Recommendations on screening for depression in adults. *CMAJ*. 2013;185(9):775-782. doi:10.1503/cmaj.130403

2.	Di Saverio S, Bassi M, Smerieri N, et al. Diagnosis and treatment of perforated or bleeding peptic ulcers: 2013 WSES position paper. *World J Emerg Surg*. 2014;9:45. doi:10.1186/1749-7922-9-45

3.	TheTask Force on cardiac pacing and resynchronization therapy of the European Society of Cardiology (ESC). Developed in collaboration with the European Heart Rhythm Association (EHRA), Brignole M, Auricchio A, et al. 2013 ESC Guidelines on cardiac pacing and cardiac resynchronization therapy. *Rev Esp Cardiol (Engl Ed)*. 2014;67(1):58. doi:10.1016/j.rec.2013.11.003

4.	Pedersen CT, Kay GN, Kalman J, et al. EHRA/HRS/APHRS expert consensus on ventricular arrhythmias. *Europace*. 2014;16(9):1257-1283. doi:10.1093/europace/euu194

5.	Taylor J. 2013 ESH/ESC guidelines for the management of arterial hypertension. *Eur Heart J*. 2013;34(28):2108-2109.

6.	Smith SC, Benjamin EJ, Bonow RO, et al. AHA/ACCF Secondary Prevention and Risk Reduction Therapy for Patients with Coronary and other Atherosclerotic Vascular Disease: 2011 update: a guideline from the American Heart Association and American College of Cardiology Foundation. *Circulation*. 2011;124(22):2458-2473. doi:10.1161/CIR.0b013e318235eb4d

7.	Standards of medical care in diabetes--2015: summary of revisions. *Diabetes Care*. 2015;38 Suppl:S4. doi:10.2337/dc15-S003

8.	Meschia JF, Bushnell C, Boden-Albala B, et al. Guidelines for the primary prevention of stroke: a statement for healthcare professionals from the American Heart Association/American Stroke Association. *Stroke*. 2014;45(12):3754-3832. doi:10.1161/STR.0000000000000046

9.	Eccles S, Pincus C, Higgins B, Woodhead M, Guideline Development Group. Diagnosis and management of community and hospital acquired pneumonia in adults: summary of NICE guidance. *BMJ*. 2014;349:g6722. doi:10.1136/bmj.g6722

10.	Sarnak MJ, Bloom R, Muntner P, et al. KDOQI US commentary on the 2013 KDIGO Clinical Practice Guideline for Lipid Management in CKD. *Am J Kidney Dis*. 2015;65(3):354-366. doi:10.1053/j.ajkd.2014.10.005

11.	Runyon BA, AASLD. Introduction to the revised American Association for the Study of Liver Diseases Practice Guideline management of adult patients with ascites due to cirrhosis 2012. *Hepatology*. 2013;57(4):1651-1653. doi:10.1002/hep.26359

12.	Singh JA, Furst DE, Bharat A, et al. 2012 update of the 2008 American College of Rheumatology recommendations for the use of disease-modifying antirheumatic drugs and biologic agents in the treatment of rheumatoid arthritis. *Arthritis Care Res (Hoboken)*. 2012;64(5):625-639. doi:10.1002/acr.21641

13.	Ohta K, Ichinose M, Nagase H, et al. Japanese Guideline for Adult Asthma 2014. *Allergol Int*. 2014;63(3):293-333.

14.	Welborn TA. Practice guidelines for hypothyroidism in adults: clinical issues and the controversy about combination therapy. *Endocr Pract*. 2013;19(6):1062-1064.

15.	Cosman F, de Beur SJ, LeBoff MS, et al. Clinician’s Guide to Prevention and Treatment of Osteoporosis. *Osteoporos Int*. 2014;25(10):2359-2381. doi:10.1007/s00198-014-2794-2

16.	Goddard AF, James MW, McIntyre AS, Scott BB, British Society of Gastroenterology. Guidelines for the management of iron deficiency anaemia. *Gut*. 2011;60(10):1309-1316. doi:10.1136/gut.2010.228874

## eTable 2 Baseline characteristics

| Variables | Total  (n = 4872) | Control  (n = 3582) | Recent-onset AF  (n = 1290) | Statistic | *P* |  |
| --- | --- | --- | --- | --- | --- | --- |
|  |  |  |  |  |  |  |
| Age, M (Q₁, Q₃) | 68.00 (62.00, 74.00) | 68.00 (61.00, 74.00) | 69.00 (63.00, 76.00) | Z=-5.81 | <.001 |  |
| Temperature, M (Q₁, Q₃) | 36.40 (36.20, 36.50) | 36.40 (36.20, 36.50) | 36.40 (36.26, 36.50) | Z=-1.59 | 0.112 |  |
| HR, M (Q₁, Q₃) | 74.00 (67.92, 81.00) | 74.00 (67.81, 80.14) | 75.00 (68.00, 82.00) | Z=-2.58 | 0.010 |  |
| Respiratory Rate, M (Q₁, Q₃) | 18.00 (18.00, 18.60) | 18.00 (17.80, 18.60) | 18.00 (18.00, 18.80) | Z=-2.40 | 0.016 |  |
| SBP, M (Q₁, Q₃) | 135.00 (122.00, 148.00) | 135.00 (123.00, 148.00) | 134.00 (120.00, 147.00) | Z=-2.91 | 0.004 |  |
| DBP, M (Q₁, Q₃) | 77.00 (68.00, 84.00) | 76.70 (69.00, 84.00) | 77.00 (68.00, 84.00) | Z=-0.22 | 0.824 |  |
| NLR, M (Q₁, Q₃) | 2.43 (1.96, 3.26) | 2.43 (1.88, 3.00) | 2.92 (2.22, 4.01) | Z=-14.82 | <.001 |  |
| PLR, M (Q₁, Q₃) | 130.57 (105.64, 168.43) | 130.57 (103.43, 159.78) | 141.79 (113.57, 201.41) | Z=-10.85 | <.001 |  |
| SII, M (Q₁, Q₃) | 498.79 (376.99, 717.66) | 498.79 (364.00, 648.51) | 603.57 (424.84, 902.50) | Z=-13.05 | <.001 |  |
| SIRI, M (Q₁, Q₃) | 0.92 (0.69, 1.42) | 0.92 (0.65, 1.26) | 1.18 (0.81, 1.90) | Z=-13.62 | <.001 |  |
| TyG, M (Q₁, Q₃) | 6.02 (5.73, 6.36) | 5.98 (5.72, 6.30) | 6.14 (5.78, 6.57) | Z=-9.21 | <.001 |  |
| LDH, M (Q₁, Q₃) | 163.00 (146.00, 186.00) | 163.00 (145.00, 183.00) | 168.00 (149.00, 196.27) | Z=-6.40 | <.001 |  |
| IgA, M (Q₁, Q₃) | 2.36 (1.95, 2.89) | 2.36 (1.94, 2.88) | 2.36 (1.98, 2.91) | Z=-1.24 | 0.215 |  |
| IgG, M (Q₁, Q₃) | 11.26 (10.13, 12.58) | 11.26 (10.16, 12.55) | 11.26 (10.08, 12.64) | Z=-0.17 | 0.866 |  |
| IgM, M (Q₁, Q₃) | 0.75 (0.58, 0.95) | 0.75 (0.58, 0.96) | 0.75 (0.57, 0.94) | Z=-1.73 | 0.084 |  |
| Baso Count, M (Q₁, Q₃) | 0.03 (0.02, 0.04) | 0.03 (0.02, 0.04) | 0.03 (0.02, 0.04) | Z=-1.31 | 0.189 |  |
| Baso Ratio, M (Q₁, Q₃) | 0.50 (0.40, 0.70) | 0.50 (0.40, 0.70) | 0.50 (0.30, 0.60) | Z=-3.74 | <.001 |  |
| Eos Count, M (Q₁, Q₃) | 0.12 (0.07, 0.18) | 0.12 (0.08, 0.18) | 0.12 (0.07, 0.18) | Z=-2.12 | 0.034 |  |
| Eos Ratio, M (Q₁, Q₃) | 2.00 (1.20, 3.00) | 2.00 (1.30, 3.00) | 2.00 (1.10, 3.00) | Z=-3.49 | <.001 |  |
| WBC, M (Q₁, Q₃) | 6.04 (5.18, 7.15) | 6.04 (5.12, 7.08) | 6.16 (5.33, 7.40) | Z=-4.32 | <.001 |  |
| ALP, M (Q₁, Q₃) | 70.00 (61.00, 83.00) | 70.00 (61.31, 83.00) | 70.00 (61.00, 83.00) | Z=-0.10 | 0.924 |  |
| Casts, M (Q₁, Q₃) | 0.13 (0.00, 0.27) | 0.13 (0.00, 0.27) | 0.13 (0.00, 0.28) | Z=-2.41 | 0.016 |  |
| HbA1c, M (Q₁, Q₃) | 6.10 (5.75, 6.60) | 6.10 (5.70, 6.60) | 6.10 (5.75, 6.70) | Z=-2.37 | 0.018 |  |
| RBC, M (Q₁, Q₃) | 4.27 (3.83, 4.56) | 4.27 (3.88, 4.57) | 4.20 (3.67, 4.55) | Z=-4.72 | <.001 |  |
| RDW SD, M (Q₁, Q₃) | 42.40 (40.75, 45.10) | 42.30 (40.70, 44.80) | 43.10 (41.10, 46.50) | Z=-6.07 | <.001 |  |
| Hematocrit, M (Q₁, Q₃) | 38.70 (34.90, 41.20) | 38.70 (35.40, 41.20) | 37.90 (33.90, 40.87) | Z=-4.66 | <.001 |  |
| MCV, M (Q₁, Q₃) | 90.70 (88.89, 93.50) | 90.70 (88.90, 93.20) | 90.70 (88.60, 94.10) | Z=-2.14 | 0.033 |  |
| FDP, M (Q₁, Q₃) | 2.60 (2.50, 2.60) | 2.60 (2.50, 2.60) | 2.60 (2.50, 2.70) | Z=-3.23 | 0.001 |  |
| CYFRA21, M (Q₁, Q₃) | 2.58 (2.02, 3.33) | 2.56 (2.01, 3.29) | 2.60 (2.08, 3.41) | Z=-2.05 | 0.041 |  |
| Bac Count, M (Q₁, Q₃) | 26.80 (8.20, 154.95) | 26.10 (7.20, 152.70) | 27.80 (9.10, 167.60) | Z=-1.67 | 0.095 |  |
| PDW, M (Q₁, Q₃) | 11.60 (10.70, 12.90) | 11.60 (10.80, 12.80) | 11.60 (10.50, 12.90) | Z=-1.01 | 0.313 |  |
| Hemoglobin, M (Q₁, Q₃) | 131.00 (117.00, 139.00) | 131.00 (118.00, 140.00) | 128.00 (112.00, 139.00) | Z=-5.08 | <.001 |  |
| C3, M (Q₁, Q₃) | 1.03 (0.90, 1.16) | 1.03 (0.91, 1.17) | 1.03 (0.86, 1.13) | Z=-4.28 | <.001 |  |
| C4, M (Q₁, Q₃) | 0.27 (0.22, 0.32) | 0.27 (0.23, 0.32) | 0.27 (0.22, 0.32) | Z=-2.14 | 0.032 |  |
| Ph, M (Q₁, Q₃) | 6.00 (5.50, 6.50) | 6.00 (5.50, 6.50) | 6.00 (5.50, 6.50) | Z=-1.94 | 0.052 |  |
| Magnesium, M (Q₁, Q₃) | 0.90 (0.85, 0.95) | 0.90 (0.85, 0.95) | 0.90 (0.85, 0.95) | Z=-1.97 | 0.049 |  |
| Gender, n(%) |  |  |  | χ²=0.92 | 0.338 |  |
| 1 | 2486 (51.03) | 1813 (50.61) | 673 (52.17) |  |  |  |
| 2 | 2386 (48.97) | 1769 (49.39) | 617 (47.83) |  |  |  |
| Chronic Kidney Disease, n(%) |  |  |  | χ²=8.68 | 0.003 |  |
| 0 | 4739 (97.27) | 3499 (97.68) | 1240 (96.12) |  |  |  |
| 1 | 133 (2.73) | 83 (2.32) | 50 (3.88) |  |  |  |
| Bradycardia, n(%) |  |  |  | χ²=1.28 | 0.257 |  |
| 0 | 4608 (94.58) | 3380 (94.36) | 1228 (95.19) |  |  |  |
| 1 | 264 (5.42) | 202 (5.64) | 62 (4.81) |  |  |  |
| Arrhythmia, n(%) |  |  |  | χ²=0.00 | 0.993 |  |
| 0 | 3932 (80.71) | 2891 (80.71) | 1041 (80.70) |  |  |  |
| 1 | 940 (19.29) | 691 (19.29) | 249 (19.30) |  |  |  |
| Anxiety, n(%) |  |  |  | χ²=1.39 | 0.238 |  |
| 0 | 4671 (95.87) | 3427 (95.67) | 1244 (96.43) |  |  |  |
| 1 | 201 (4.13) | 155 (4.33) | 46 (3.57) |  |  |  |
| Depression, n(%) |  |  |  | χ²=0.18 | 0.673 |  |
| 0 | 4604 (94.50) | 3382 (94.42) | 1222 (94.73) |  |  |  |
| 1 | 268 (5.50) | 200 (5.58) | 68 (5.27) |  |  |  |
| Tumor, n(%) |  |  |  | χ²=1.62 | 0.203 |  |
| 0 | 4550 (93.39) | 3355 (93.66) | 1195 (92.64) |  |  |  |
| 1 | 322 (6.61) | 227 (6.34) | 95 (7.36) |  |  |  |
| Hypertension, n(%) |  |  |  | χ²=11.14 | <.001 |  |
| 0 | 1236 (25.37) | 864 (24.12) | 372 (28.84) |  |  |  |
| 1 | 3636 (74.63) | 2718 (75.88) | 918 (71.16) |  |  |  |
| Coronary Heart Disease, n(%) |  |  |  | χ²=0.08 | 0.776 |  |
| 0 | 775 (15.91) | 573 (16.00) | 202 (15.66) |  |  |  |
| 1 | 4097 (84.09) | 3009 (84.00) | 1088 (84.34) |  |  |  |
| Diabetes, n(%) |  |  |  | χ²=9.68 | 0.002 |  |
| 0 | 3654 (75.00) | 2728 (76.16) | 926 (71.78) |  |  |  |
| 1 | 1218 (25.00) | 854 (23.84) | 364 (28.22) |  |  |  |
| Stroke, n(%) |  |  |  | χ²=33.16 | <.001 |  |
| 0 | 3676 (75.45) | 2779 (77.58) | 897 (69.53) |  |  |  |
| 1 | 1196 (24.55) | 803 (22.42) | 393 (30.47) |  |  |  |
| COPD, n(%) |  |  |  | χ²=0.17 | 0.678 |  |
| 0 | 4707 (96.61) | 3463 (96.68) | 1244 (96.43) |  |  |  |
| 1 | 165 (3.39) | 119 (3.32) | 46 (3.57) |  |  |  |
| Pneumonia, n(%) |  |  |  | χ²=36.63 | <.001 |  |
| 0 | 4148 (85.14) | 3116 (86.99) | 1032 (80.00) |  |  |  |
| 1 | 724 (14.86) | 466 (13.01) | 258 (20.00) |  |  |  |
| Cirrhosis, n(%) |  |  |  | χ²=0.02 | 0.900 |  |
| 0 | 4858 (99.71) | 3571 (99.69) | 1287 (99.77) |  |  |  |
| 1 | 14 (0.29) | 11 (0.31) | 3 (0.23) |  |  |  |
| Rheumatoid Arthritis, n(%) |  |  |  | χ²=0.02 | 0.894 |  |
| 0 | 4806 (98.65) | 3533 (98.63) | 1273 (98.68) |  |  |  |
| 1 | 66 (1.35) | 49 (1.37) | 17 (1.32) |  |  |  |
| Asthma, n(%) |  |  |  | χ²=0.62 | 0.429 |  |
| 0 | 4800 (98.52) | 3532 (98.60) | 1268 (98.29) |  |  |  |
| 1 | 72 (1.48) | 50 (1.40) | 22 (1.71) |  |  |  |
| Thyroid Disease, n(%) |  |  |  | χ²=0.00 | 0.969 |  |
| 0 | 3605 (73.99) | 2651 (74.01) | 954 (73.95) |  |  |  |
| 1 | 1267 (26.01) | 931 (25.99) | 336 (26.05) |  |  |  |
| Osteoporosis, n(%) |  |  |  | χ²=2.08 | 0.150 |  |
| 0 | 4629 (95.01) | 3413 (95.28) | 1216 (94.26) |  |  |  |
| 1 | 243 (4.99) | 169 (4.72) | 74 (5.74) |  |  |  |
| Anemia, n(%) |  |  |  | χ²=62.08 | <.001 |  |
| 0 | 4494 (92.24) | 3369 (94.05) | 1125 (87.21) |  |  |  |
| 1 | 378 (7.76) | 213 (5.95) | 165 (12.79) |  |  |  |
| Z: Mann-Whitney test, χ²: Chi-square test | | | | | |  |
| M: Median, Q₁: 1st Quartile, Q₃: 3 rd Quartile | | | | | |  |

## eTable 4 Classification metrics of nine machine-learning models in the training, internal test, and external validation set

| **Metrics** | **ANN** | **Decision Tree** | **Gradient Boosting** | **KNN** | **LightGBM** | **Random Forest** | **SVM** | **Extra Trees** | **XGBoost** |
| --- | --- | --- | --- | --- | --- | --- | --- | --- | --- |
| **Training set** | | | | | | | | | |
| Accuracy | 0.739 | 0.651 | 0.744 | 0.740 | 0.683 | 0.705 | 0.673 | **0.708** | 0.757 |
| Sensitivity | 0.178 | 0.630 | 0.054 | 0.094 | 0.604 | 0.550 | 0.505 | **0.575** | 0.171 |
| Specificity | 0.941 | 0.659 | 0.992 | 0.972 | 0.712 | 0.760 | 0.734 | **0.756** | 0.968 |
| Positive predictive value | 0.521 | 0.399 | 0.710 | 0.548 | 0.430 | 0.453 | 0.406 | **0.459** | 0.661 |
| Negative predictive value | 0.761 | 0.832 | 0.833 | 0.749 | 0.833 | 0.824 | 0.804 | **0.854** | 0.764 |
| F1 score | 0.266 | 0.489 | 0.101 | 0.161 | 0.502 | 0.497 | 0.450 | **0.510** | 0.271 |
| Kappa score | 0.151 | 0.244 | 0.066 | 0.090 | 0.280 | 0.291 | 0.221 | **0.306** | 0.182 |
| **Internal test set** | | | | | | | | | |
| Accuracy | 0.739 | 0.625 | 0.739 | 0.739 | 0.650 | 0.673 | 0.667 | **0.674** | 0.738 |
| Sensitivity | 0.158 | 0.556 | 0.047 | 0.070 | 0.509 | 0.460 | 0.442 | **0.478** | 0.116 |
| Specificity | 0.948 | 0.650 | 0.988 | 0.980 | 0.700 | 0.750 | 0.748 | **0.745** | 0.962 |
| Positive predictive value | 0.521 | 0.364 | 0.581 | 0.563 | 0.380 | 0.398 | 0.387 | **0.403** | 0.523 |
| Negative predictive value | 0.758 | 0.803 | 0.799 | 0.745 | 0.799 | 0.794 | 0.788 | **0.785** | 0.751 |
| F1 score | 0.242 | 0.440 | 0.086 | 0.124 | 0.435 | 0.427 | 0.413 | **0.437** | 0.190 |
| Kappa score | 0.136 | 0.176 | 0.049 | 0.070 | 0.189 | 0.200 | 0.182 | **0.211** | 0.104 |
| **External validation set** | | | | | | | | | |
| Accuracy | 0.635 | 0.585 | 0.729 | 0.740 | 0.599 | 0.718 | 0.520 | **0.698** | 0.693 |
| Sensitivity | 0.714 | 0.779 | 0.078 | 0.234 | 0.714 | 0.143 | 0.792 | **0.753** | 0.260 |
| Specificity | 0.605 | 0.510 | 0.980 | 0.935 | 0.555 | 0.940 | 0.415 | **0.675** | 0.860 |
| Positive predictive value | 0.410 | 0.380 | 0.600 | 0.581 | 0.382 | 0.478 | 0.343 | **0.472** | 0.417 |
| Negative predictive value | 0.846 | 0.857 | 0.734 | 0.760 | 0.835 | 0.740 | 0.838 | **0.877** | 0.751 |
| F1 score | 0.521 | 0.511 | 0.138 | 0.333 | 0.498 | 0.220 | 0.478 | **0.580** | 0.320 |
| Kappa score | 0.260 | 0.219 | 0.079 | 0.207 | 0.212 | 0.106 | 0.148 | **0.362** | 0.135 |

Note: Values are presented as proportions. The Extra Trees column is highlighted because it was selected as the final model. Abbreviations: ANN, artificial neural network; KNN, k-nearest neighbors; LightGBM, light gradient-boosting machine; SVM, support vector machine; XGBoost, extreme gradient boosting

## eTable 6 Validation set baseline features

| Variables | Total (n = 277) | Control(n = 200) | Recent-onset AF (n = 77) | Statistic | *P* |  |
| --- | --- | --- | --- | --- | --- | --- |
|  |  |  |  |  |  |  |
| Age, M (Q₁, Q₃) | 70.00 (65.00, 77.00) | 69.00 (65.00, 76.00) | 70.00 (62.00, 78.00) | Z=-0.30 | 0.764 |  |
| Temperature, M (Q₁, Q₃) | 36.40 (36.30, 36.50) | 36.40 (36.30, 36.50) | 36.47 (36.30, 36.50) | Z=-1.80 | 0.072 |  |
| HR, M (Q₁, Q₃) | 73.00 (66.78, 81.00) | 72.95 (65.94, 80.14) | 75.00 (68.84, 84.00) | Z=-1.58 | 0.113 |  |
| Respiratory Rate, M (Q₁, Q₃) | 18.00 (18.00, 18.60) | 18.00 (18.00, 18.40) | 18.20 (18.00, 19.00) | Z=-1.90 | 0.057 |  |
| SBP, M (Q₁, Q₃) | 137.00 (125.00, 150.00) | 137.00 (125.00, 150.00) | 136.00 (121.00, 152.00) | Z=-0.16 | 0.875 |  |
| DBP, M (Q₁, Q₃) | 76.00 (68.00, 84.00) | 75.00 (67.00, 83.00) | 76.35 (68.00, 86.00) | Z=-1.23 | 0.220 |  |
| NLR, M (Q₁, Q₃) | 2.82 (2.10, 4.07) | 2.61 (1.96, 3.64) | 3.62 (2.59, 5.01) | Z=-4.78 | **<.001** |  |
| PLR, M (Q₁, Q₃) | 134.17 (100.59, 181.45) | 130.57 (94.42, 168.16) | 145.07 (114.41, 224.32) | Z=-3.17 | **0.002** |  |
| SII, M (Q₁, Q₃) | 557.14 (372.40, 893.80) | 499.04 (354.49, 809.23) | 726.74 (498.79, 1028.10) | Z=-3.71 | **<.001** |  |
| SIRI, M (Q₁, Q₃) | 1.17 (0.85, 2.24) | 1.04 (0.70, 1.81) | 1.88 (1.07, 3.09) | Z=-4.92 | **<.001** |  |
| TyG, M (Q₁, Q₃) | 6.06 (5.77, 6.52) | 6.01 (5.67, 6.42) | 6.22 (5.88, 6.79) | Z=-2.96 | **0.003** |  |
| LDH, M (Q₁, Q₃) | 178.00 (155.00, 215.00) | 177.92 (155.00, 213.50) | 179.00 (156.39, 216.00) | Z=-0.05 | 0.963 |  |
| IgA, M (Q₁, Q₃) | 2.36 (1.88, 2.89) | 2.36 (1.88, 2.88) | 2.33 (1.90, 2.91) | Z=-0.07 | 0.948 |  |
| IgG, M (Q₁, Q₃) | 11.26 (10.14, 12.60) | 11.30 (10.33, 12.60) | 11.14 (9.68, 12.40) | Z=-1.53 | 0.126 |  |
| IgM, M (Q₁, Q₃) | 0.74 (0.56, 0.96) | 0.75 (0.56, 0.98) | 0.73 (0.56, 0.87) | Z=-0.26 | 0.796 |  |
| Baso Count, M (Q₁, Q₃) | 0.03 (0.02, 0.05) | 0.03 (0.02, 0.04) | 0.03 (0.02, 0.05) | Z=-0.10 | 0.922 |  |
| Baso Ratio, M (Q₁, Q₃) | 0.50 (0.40, 0.70) | 0.53 (0.40, 0.70) | 0.50 (0.30, 0.80) | Z=-0.71 | 0.478 |  |
| Eos Count, M (Q₁, Q₃) | 0.13 (0.09, 0.23) | 0.13 (0.09, 0.20) | 0.15 (0.09, 0.26) | Z=-1.17 | 0.244 |  |
| Eos Ratio, M (Q₁, Q₃) | 2.20 (1.40, 3.60) | 2.15 (1.40, 3.23) | 2.30 (1.50, 5.00) | Z=-1.25 | 0.213 |  |
| WBC, M (Q₁, Q₃) | 6.33 (5.30, 7.70) | 6.30 (5.28, 7.60) | 6.49 (5.32, 8.34) | Z=-0.95 | 0.344 |  |
| ALP, M (Q₁, Q₃) | 74.00 (64.75, 89.00) | 74.23 (64.28, 89.00) | 74.00 (65.00, 87.21) | Z=-0.15 | 0.881 |  |
| Casts, M (Q₁, Q₃) | 0.14 (0.05, 0.30) | 0.14 (0.00, 0.28) | 0.14 (0.13, 0.42) | Z=-1.56 | 0.118 |  |
| HbA1c, M (Q₁, Q₃) | 6.10 (5.75, 6.50) | 6.10 (5.75, 6.50) | 6.10 (5.75, 6.80) | Z=-0.31 | 0.754 |  |
| RBC, M (Q₁, Q₃) | 4.05 (3.57, 4.38) | 4.12 (3.68, 4.41) | 3.75 (3.26, 4.29) | Z=-2.61 | **0.009** |  |
| RDW SD, M (Q₁, Q₃) | 44.80 (42.30, 50.40) | 44.35 (42.00, 47.92) | 48.00 (43.40, 53.90) | Z=-3.17 | **0.002** |  |
| Hematocrit, M (Q₁, Q₃) | 37.10 (32.60, 40.10) | 37.60 (33.58, 40.42) | 35.50 (30.47, 38.70) | Z=-2.34 | **0.019** |  |
| MCV, M (Q₁, Q₃) | 91.80 (89.30, 95.50) | 91.61 (89.24, 95.20) | 92.00 (89.40, 97.10) | Z=-1.24 | 0.215 |  |
| FDP, M (Q₁, Q₃) | 2.60 (2.50, 2.90) | 2.60 (2.50, 2.90) | 2.70 (2.60, 2.90) | Z=-2.79 | **0.005** |  |
| CYFRA21, M (Q₁, Q₃) | 2.90 (2.31, 3.79) | 2.89 (2.27, 3.86) | 2.98 (2.38, 3.66) | Z=-0.65 | 0.518 |  |
| Bac Count, M (Q₁, Q₃) | 39.90 (9.70, 179.90) | 35.75 (9.30, 180.03) | 52.40 (19.50, 168.45) | Z=-1.35 | 0.177 |  |
| PDW, M (Q₁, Q₃) | 11.90 (11.10, 13.60) | 11.85 (11.00, 13.53) | 12.50 (11.40, 14.10) | Z=-1.86 | 0.063 |  |
| Hemoglobin, M (Q₁, Q₃) | 124.00 (105.00, 135.00) | 127.50 (108.75, 136.00) | 117.00 (100.00, 131.00) | Z=-2.35 | **0.019** |  |
| C3, M (Q₁, Q₃) | 0.96 (0.84, 1.07) | 0.97 (0.85, 1.09) | 0.93 (0.83, 1.03) | Z=-2.11 | **0.035** |  |
| C4, M (Q₁, Q₃) | 0.27 (0.23, 0.32) | 0.27 (0.23, 0.32) | 0.27 (0.23, 0.32) | Z=-0.14 | 0.888 |  |
| Ph, M (Q₁, Q₃) | 6.00 (5.50, 6.60) | 6.00 (5.50, 6.61) | 6.00 (5.50, 6.55) | Z=-0.01 | 0.991 |  |
| Magnesium, M (Q₁, Q₃) | 1.06 (1.04, 1.10) | 1.06 (1.04, 1.09) | 1.06 (1.04, 1.12) | Z=-1.01 | 0.313 |  |
| Gender, n(%) |  |  |  | χ²=0.28 | 0.595 |  |
| 1 | 126 (45.49) | 89 (44.50) | 37 (48.05) |  |  |  |
| 2 | 151 (54.51) | 111 (55.50) | 40 (51.95) |  |  |  |
| Bradycardia, n(%) |  |  |  | χ²=0.98 | 0.322 |  |
| 0 | 262 (94.58) | 187 (93.50) | 75 (97.40) |  |  |  |
| 1 | 15 (5.42) | 13 (6.50) | 2 (2.60) |  |  |  |
| Arrhythmia, n(%) |  |  |  | χ²=5.45 | **0.020** |  |
| 0 | 237 (85.56) | 165 (82.50) | 72 (93.51) |  |  |  |
| 1 | 40 (14.44) | 35 (17.50) | 5 (6.49) |  |  |  |
| Anxiety, n(%) |  |  |  | χ²=0.59 | 0.443 |  |
| 0 | 271 (97.83) | 197 (98.50) | 74 (96.10) |  |  |  |
| 1 | 6 (2.17) | 3 (1.50) | 3 (3.90) |  |  |  |
| Depression, n(%) |  |  |  | χ²=0.37 | 0.545 |  |
| 0 | 261 (94.22) | 190 (95.00) | 71 (92.21) |  |  |  |
| 1 | 16 (5.78) | 10 (5.00) | 6 (7.79) |  |  |  |
| Tumor, n(%) |  |  |  | χ²=2.05 | 0.153 |  |
| 0 | 255 (92.06) | 187 (93.50) | 68 (88.31) |  |  |  |
| 1 | 22 (7.94) | 13 (6.50) | 9 (11.69) |  |  |  |
| Hypertension, n(%) |  |  |  | χ²=0.06 | 0.805 |  |
| 0 | 62 (22.38) | 44 (22.00) | 18 (23.38) |  |  |  |
| 1 | 215 (77.62) | 156 (78.00) | 59 (76.62) |  |  |  |
| Coronary Heart Disease, n(%) |  |  |  | χ²=0.05 | 0.819 |  |
| 0 | 27 (9.75) | 20 (10.00) | 7 (9.09) |  |  |  |
| 1 | 250 (90.25) | 180 (90.00) | 70 (90.91) |  |  |  |
| Diabetes, n(%) |  |  |  | χ²=1.90 | 0.168 |  |
| 0 | 220 (79.42) | 163 (81.50) | 57 (74.03) |  |  |  |
| 1 | 57 (20.58) | 37 (18.50) | 20 (25.97) |  |  |  |
| Stroke, n(%) |  |  |  | χ²=1.41 | 0.235 |  |
| 0 | 228 (82.31) | 168 (84.00) | 60 (77.92) |  |  |  |
| 1 | 49 (17.69) | 32 (16.00) | 17 (22.08) |  |  |  |
| COPD, n(%) |  |  |  | χ²=0.00 | 1.000 |  |
| 0 | 269 (97.11) | 194 (97.00) | 75 (97.40) |  |  |  |
| 1 | 8 (2.89) | 6 (3.00) | 2 (2.60) |  |  |  |
| Pneumonia, n(%) |  |  |  | χ²=3.75 | 0.053 |  |
| 0 | 219 (79.06) | 164 (82.00) | 55 (71.43) |  |  |  |
| 1 | 58 (20.94) | 36 (18.00) | 22 (28.57) |  |  |  |
| Chronic Kidney Disease, n(%) |  |  |  | χ²=0.98 | 0.321 |  |
| 0 | 260 (93.86) | 190 (95.00) | 70 (90.91) |  |  |  |
| 1 | 17 (6.14) | 10 (5.00) | 7 (9.09) |  |  |  |
| Rheumatoid Arthritis, n(%) |  |  |  | χ²=0.00 | 1.000 |  |
| 0 | 273 (98.56) | 197 (98.50) | 76 (98.70) |  |  |  |
| 1 | 4 (1.44) | 3 (1.50) | 1 (1.30) |  |  |  |
| Asthma, n(%) |  |  |  | χ²=0.00 | 1.000 |  |
| 0 | 273 (98.56) | 197 (98.50) | 76 (98.70) |  |  |  |
| 1 | 4 (1.44) | 3 (1.50) | 1 (1.30) |  |  |  |
| Thyroid Disease, n(%) |  |  |  | χ²=0.19 | 0.666 |  |
| 0 | 224 (80.87) | 163 (81.50) | 61 (79.22) |  |  |  |
| 1 | 53 (19.13) | 37 (18.50) | 16 (20.78) |  |  |  |
| Osteoporosis, n(%) |  |  |  | χ²=0.00 | 1.000 |  |
| 0 | 266 (96.03) | 192 (96.00) | 74 (96.10) |  |  |  |
| 1 | 11 (3.97) | 8 (4.00) | 3 (3.90) |  |  |  |
| Anemia, n(%) |  |  |  | χ²=11.80 | **<.001** |  |
| 0 | 240 (86.64) | 182 (91.00) | 58 (75.32) |  |  |  |
| 1 | 37 (13.36) | 18 (9.00) | 19 (24.68) |  |  |  |
| Z: Mann-Whitney test, χ²: Chi-square test | | | | | |  |
| M: Median, Q₁: 1st Quartile, Q₃: 3rd Quartile | | | | | |  |

## eTable 7 Discrimination, calibration, and reclassification metrics of nine machine-learning models in the training, internal test, and external validation set

| **Dataset** | **Model** | **AUC (95% CI)** | **Brier score** | **Calibration- in-the-large** | **Calibration slope** | **O/E ratio** | **Continuous NRI, % (95% CI); P** | **IDI, % (95% CI); P** |
| --- | --- | --- | --- | --- | --- | --- | --- | --- |
| **Training set** | **Random Forest** | 0.734 (0.715, 0.753) | 0.211 | -0.951 | 1.611 | 0.566 | 0.00 (0.00, 0.00); P=1.000 | -3.20 (-3.58, -2.81); P=0.001 |
|  | **LightGBM** | 0.722 (0.703, 0.741) | 0.214 | -0.994 | 1.396 | 0.558 | -1.36 (-1.99, -0.80); P=0.001 | -3.95 (-4.43, -3.45); P=0.001 |
|  | **Gradient Boosting** | 0.686 (0.664, 0.706) | 0.178 | -0.000 | 1.382 | 1.000 | 10.07 (2.47, 17.53); P=0.008 | 0.56 (0.25, 0.87); P=0.001 |
|  | **XGBoost** | 0.724 (0.705, 0.742) | 0.171 | -0.001 | 1.311 | 0.999 | -44.33 (-51.57, -36.87); P=0.001 | -2.96 (-3.38, -2.54); P=0.001 |
|  | **SVM** | 0.668 (0.647, 0.688) | 0.180 | -0.000 | 1.099 | 1.000 | -3.29 (-10.79, 4.04); P=0.408 | 0.38 (-0.07, 0.84); P=0.098 |
|  | **KNN** | 0.675 (0.654, 0.695) | 0.180 | 0.086 | 1.143 | 1.062 | 0.22 (-7.35, 7.28); P=0.950 | 0.53 (0.09, 0.94); P=0.022 |
|  | **Decision Tree** | 0.687 (0.667, 0.707) | 0.220 | -1.021 | 1.000 | 0.560 | -2.48 (-3.88, -1.02); P=0.001 | -4.21 (-5.02, -3.44); P=0.001 |
|  | **ET** | **0.853 (0.832, 0.874)** | **0.180** | **-0.002** | **1.095** | **0.998** | **Reference** | **Reference** |
|  | **ANN** | 0.638 (0.616, 0.662) | 0.184 | -0.062 | 0.722 | 0.961 | -3.04 (-10.47, 4.52); P=0.401 | -0.92 (-1.44, -0.43); P=0.001 |
| **Internal test set** | **Random Forest** | 0.641 (0.609, 0.674) | 0.224 | -0.921 | 1.001 | 0.573 | 0.00 (0.00, 0.00); P=1.000 | -0.60 (-1.12, -0.04); P=0.031 |
|  | **LightGBM** | 0.636 (0.603, 0.669) | 0.228 | -0.967 | 0.846 | 0.564 | 0.14 (-0.74, 1.55); P=0.967 | -1.15 (-1.92, -0.43); P=0.003 |
|  | **Gradient Boosting** | 0.634 (0.602, 0.666) | 0.185 | 0.022 | 1.040 | 1.016 | 10.40 (-1.20, 21.54); P=0.080 | 0.66 (0.22, 1.10); P=0.004 |
|  | **XGBoost** | 0.635 (0.604, 0.669) | 0.185 | 0.035 | 0.804 | 1.024 | -21.19 (-32.69, -9.45); P=0.001 | -0.67 (-1.28, -0.05); P=0.034 |
|  | **SVM** | 0.640 (0.607, 0.673) | 0.185 | 0.042 | 0.897 | 1.029 | -5.93 (-16.91, 5.06); P=0.335 | 0.06 (-0.66, 0.79); P=0.828 |
|  | **KNN** | 0.620 (0.584, 0.654) | 0.187 | 0.125 | 0.876 | 1.092 | 8.81 (-2.24, 20.09); P=0.122 | 0.69 (0.07, 1.32); P=0.031 |
|  | **Decision Tree** | 0.626 (0.594, 0.657) | 0.234 | -1.000 | 0.426 | 0.563 | -0.53 (-2.65, 2.36); P=0.770 | -1.70 (-2.94, -0.56); P=0.003 |
|  | **ET** | **0.636 (0.602, 0.670)** | **0.185** | **0.035** | **0.919** | **1.025** | **Reference** | **Reference** |
|  | **ANN** | 0.607 (0.573, 0.641) | 0.189 | -0.011 | 0.605 | 0.993 | -4.30 (-16.08, 7.01); P=0.464 | -0.82 (-1.58, -0.09); P=0.027 |
| **External validation set** | **Random Forest** | 0.706 (0.635, 0.770) | 0.237 | -1.099 | 1.377 | 0.537 | 0.00 (0.00, 0.00); P=1.000 | -1.16 (-2.63, 0.24); P=0.104 |
|  | **LightGBM** | 0.694 (0.623, 0.762) | 0.247 | -1.173 | 1.141 | 0.524 | -1.00 (-3.00, 0.00); P=0.741 | -1.46 (-3.14, 0.31); P=0.112 |
|  | **Gradient Boosting** | 0.665 (0.593, 0.735) | 0.188 | -0.140 | 1.157 | 0.911 | 27.49 (0.71, 53.87); P=0.040 | 2.01 (0.73, 3.32); P=0.001 |
|  | **XGBoost** | 0.694 (0.623, 0.761) | 0.184 | -0.187 | 1.056 | 0.886 | -12.69 (-38.08, 12.90); P=0.328 | -0.97 (-2.39, 0.52); P=0.178 |
|  | **SVM** | 0.650 (0.579, 0.720) | 0.191 | -0.168 | 0.974 | 0.895 | 18.68 (-5.71, 43.46); P=0.167 | 2.00 (0.11, 3.86); P=0.037 |
|  | **KNN** | 0.670 (0.600, 0.740) | 0.188 | -0.083 | 1.001 | 0.946 | 1.98 (-25.31, 27.45); P=0.889 | 1.55 (0.07, 3.15); P=0.044 |
|  | **Decision Tree** | 0.677 (0.602, 0.749) | 0.255 | -1.249 | 0.812 | 0.518 | -5.00 (-10.00, -1.00); P=0.011 | -2.38 (-5.16, 0.30); P=0.069 |
|  | **ET** | **0.766 (0.696, 0.837)** | **0.188** | **-0.176** | **0.936** | **0.893** | **Reference** | **Reference** |
|  | **ANN** | 0.650 (0.573, 0.724) | 0.195 | -0.241 | 0.630 | 0.866 | -3.09 (-29.87, 21.89); P=0.871 | -1.78 (-3.74, 0.18); P=0.077 |

Note: Values are presented as proportions. The Extra Trees column is highlighted because it was selected as the final model. Abbreviations: ANN, artificial neural network; KNN, k-nearest neighbors; LightGBM, light gradient-boosting machine; SVM, support vector machine; XGBoost, extreme gradient boosting..

## eTable 8 Segmented Regression–Derived Change Points and Multiple-Testing–Adjusted Davies Tests for NLR and TyG in Relation to Recent-Onset Atrial Fibrillation

| **Marker** | **Change point** | **Segment** | **OR (95% CI)** | **Segment P value** | **AIC, linear** | **AIC, segmented** | **Davies test P value** | **Holm-adjusted P value** | **BH-adjusted P value** |
| --- | --- | --- | --- | --- | --- | --- | --- | --- | --- |
| **NLR** | 4.24 | Below breakpoint | 1.97 (1.79–2.17) | <0.001 | 5413.184 | 5378.819 | 2.38 × 10⁻⁸ | 1.43 × 10⁻⁷ | 1.43 × 10⁻⁷ |
|  |  | Above breakpoint | 1.21 (1.04 - 1.41) | 0.013 |  |  |  |  |  |
| **TyG** | 5.91 | Below breakpoint | 0.91 (0.59–1.40) | 0.667 | 5630.846 | 5607.606 | 3.99 × 10⁻⁶ | 1.99 × 10⁻⁵ | 1.20 × 10⁻⁵ |
|  |  | Above breakpoint | 3.01 (2.49–3.62) | <0.001 |  |  |  |  |  |

**Abbreviations: AF, atrial fibrillation; AIC, Akaike information criterion; BH, Benjamini–Hochberg; NLR, neutrophil-to-lymphocyte ratio; TyG, triglyceride-glucose index.**

## eTable 9 Variance inflation factor and generalized variance inflation factor diagnostics for the mediation models

| **Model information** | | | | | **Collinearity diagnostics** | | | |
| --- | --- | --- | --- | --- | --- | --- | --- | --- |
| **Pathway** | **Model** | **Model stage** | **Role** | **Term** | **GVIF** | **Df** | **Adjusted GVIF** | **Comparable VIF** |
| TyG-NLR-AF | Original model | Mediator model | Exposure | TyG | 1.078 | 1 | 1.038 | 1.078 |
| TyG-NLR-AF | Original model | Mediator model | Covariate | Gender | 1.060 | 1 | 1.030 | 1.060 |
| TyG-NLR-AF | Original model | Mediator model | Covariate | Chronic_Kidney_Disease | 1.034 | 1 | 1.017 | 1.034 |
| TyG-NLR-AF | Original model | Mediator model | Covariate | Bradycardia | 1.124 | 1 | 1.060 | 1.124 |
| TyG-NLR-AF | Original model | Mediator model | Covariate | Arrhythmia | 1.190 | 1 | 1.091 | 1.190 |
| TyG-NLR-AF | Original model | Mediator model | Covariate | Anxiety | 1.266 | 1 | 1.125 | 1.266 |
| TyG-NLR-AF | Original model | Mediator model | Covariate | Depression | 1.240 | 1 | 1.114 | 1.240 |
| TyG-NLR-AF | Original model | Mediator model | Covariate | Tumor | 1.050 | 1 | 1.025 | 1.050 |
| TyG-NLR-AF | Original model | Mediator model | Covariate | Hypertension | 1.042 | 1 | 1.021 | 1.042 |
| TyG-NLR-AF | Original model | Mediator model | Covariate | Coronary_Heart_Disease | 1.087 | 1 | 1.043 | 1.087 |
| TyG-NLR-AF | Original model | Mediator model | Covariate | Diabetes | 1.166 | 1 | 1.080 | 1.166 |
| TyG-NLR-AF | Original model | Mediator model | Covariate | Stroke | 1.169 | 1 | 1.081 | 1.169 |
| TyG-NLR-AF | Original model | Mediator model | Covariate | COPD | 1.005 | 1 | 1.003 | 1.005 |
| TyG-NLR-AF | Original model | Mediator model | Covariate | Pneumonia | 1.064 | 1 | 1.031 | 1.064 |
| TyG-NLR-AF | Original model | Mediator model | Covariate | Cirrhosis | 1.007 | 1 | 1.004 | 1.007 |
| TyG-NLR-AF | Original model | Mediator model | Covariate | Rheumatoid_Arthritis | 1.057 | 1 | 1.028 | 1.057 |
| TyG-NLR-AF | Original model | Mediator model | Covariate | Asthma | 1.012 | 1 | 1.006 | 1.012 |
| TyG-NLR-AF | Original model | Mediator model | Covariate | Thyroid_Disease | 1.196 | 1 | 1.094 | 1.196 |
| TyG-NLR-AF | Original model | Mediator model | Covariate | Osteoporosis | 1.115 | 1 | 1.056 | 1.115 |
| TyG-NLR-AF | Original model | Mediator model | Covariate | Anemia | 1.097 | 1 | 1.047 | 1.097 |
| TyG-NLR-AF | Original model | Outcome model | Exposure | TyG | 1.543 | 1 | 1.242 | 1.543 |
| TyG-NLR-AF | Original model | Outcome model | Mediator | NLR | 1.494 | 1 | 1.222 | 1.494 |
| TyG-NLR-AF | Original model | Outcome model | Covariate | Gender | 1.065 | 1 | 1.032 | 1.065 |
| TyG-NLR-AF | Original model | Outcome model | Covariate | Chronic_Kidney_Disease | 1.040 | 1 | 1.020 | 1.040 |
| TyG-NLR-AF | Original model | Outcome model | Covariate | Bradycardia | 1.116 | 1 | 1.057 | 1.116 |
| TyG-NLR-AF | Original model | Outcome model | Covariate | Arrhythmia | 1.181 | 1 | 1.087 | 1.181 |
| TyG-NLR-AF | Original model | Outcome model | Covariate | Anxiety | 1.246 | 1 | 1.116 | 1.246 |
| TyG-NLR-AF | Original model | Outcome model | Covariate | Depression | 1.224 | 1 | 1.106 | 1.224 |
| TyG-NLR-AF | Original model | Outcome model | Covariate | Tumor | 1.051 | 1 | 1.025 | 1.051 |
| TyG-NLR-AF | Original model | Outcome model | Covariate | Hypertension | 1.051 | 1 | 1.025 | 1.051 |
| TyG-NLR-AF | Original model | Outcome model | Covariate | Coronary_Heart_Disease | 1.088 | 1 | 1.043 | 1.088 |
| TyG-NLR-AF | Original model | Outcome model | Covariate | Diabetes | 1.186 | 1 | 1.089 | 1.186 |
| TyG-NLR-AF | Original model | Outcome model | Covariate | Stroke | 1.197 | 1 | 1.094 | 1.197 |
| TyG-NLR-AF | Original model | Outcome model | Covariate | COPD | 1.007 | 1 | 1.004 | 1.007 |
| TyG-NLR-AF | Original model | Outcome model | Covariate | Pneumonia | 1.064 | 1 | 1.031 | 1.064 |
| TyG-NLR-AF | Original model | Outcome model | Covariate | Cirrhosis | 1.006 | 1 | 1.003 | 1.006 |
| TyG-NLR-AF | Original model | Outcome model | Covariate | Rheumatoid_Arthritis | 1.057 | 1 | 1.028 | 1.057 |
| TyG-NLR-AF | Original model | Outcome model | Covariate | Asthma | 1.012 | 1 | 1.006 | 1.012 |
| TyG-NLR-AF | Original model | Outcome model | Covariate | Thyroid_Disease | 1.200 | 1 | 1.095 | 1.200 |
| TyG-NLR-AF | Original model | Outcome model | Covariate | Osteoporosis | 1.118 | 1 | 1.057 | 1.118 |
| TyG-NLR-AF | Original model | Outcome model | Covariate | Anemia | 1.099 | 1 | 1.048 | 1.099 |
| NLR-TyG-AF | Original model | Mediator model | Exposure | NLR | 1.053 | 1 | 1.026 | 1.053 |
| NLR-TyG-AF | Original model | Mediator model | Covariate | Gender | 1.057 | 1 | 1.028 | 1.057 |
| NLR-TyG-AF | Original model | Mediator model | Covariate | Chronic_Kidney_Disease | 1.035 | 1 | 1.017 | 1.035 |
| NLR-TyG-AF | Original model | Mediator model | Covariate | Bradycardia | 1.124 | 1 | 1.060 | 1.124 |
| NLR-TyG-AF | Original model | Mediator model | Covariate | Arrhythmia | 1.188 | 1 | 1.090 | 1.188 |
| NLR-TyG-AF | Original model | Mediator model | Covariate | Anxiety | 1.266 | 1 | 1.125 | 1.266 |
| NLR-TyG-AF | Original model | Mediator model | Covariate | Depression | 1.240 | 1 | 1.113 | 1.240 |
| NLR-TyG-AF | Original model | Mediator model | Covariate | Tumor | 1.050 | 1 | 1.025 | 1.050 |
| NLR-TyG-AF | Original model | Mediator model | Covariate | Hypertension | 1.041 | 1 | 1.020 | 1.041 |
| NLR-TyG-AF | Original model | Mediator model | Covariate | Coronary_Heart_Disease | 1.085 | 1 | 1.042 | 1.085 |
| NLR-TyG-AF | Original model | Mediator model | Covariate | Diabetes | 1.145 | 1 | 1.070 | 1.145 |
| NLR-TyG-AF | Original model | Mediator model | Covariate | Stroke | 1.163 | 1 | 1.078 | 1.163 |
| NLR-TyG-AF | Original model | Mediator model | Covariate | COPD | 1.005 | 1 | 1.002 | 1.005 |
| NLR-TyG-AF | Original model | Mediator model | Covariate | Pneumonia | 1.069 | 1 | 1.034 | 1.069 |
| NLR-TyG-AF | Original model | Mediator model | Covariate | Cirrhosis | 1.007 | 1 | 1.003 | 1.007 |
| NLR-TyG-AF | Original model | Mediator model | Covariate | Rheumatoid_Arthritis | 1.056 | 1 | 1.028 | 1.056 |
| NLR-TyG-AF | Original model | Mediator model | Covariate | Asthma | 1.012 | 1 | 1.006 | 1.012 |
| NLR-TyG-AF | Original model | Mediator model | Covariate | Thyroid_Disease | 1.190 | 1 | 1.091 | 1.190 |
| NLR-TyG-AF | Original model | Mediator model | Covariate | Osteoporosis | 1.114 | 1 | 1.056 | 1.114 |
| NLR-TyG-AF | Original model | Mediator model | Covariate | Anemia | 1.101 | 1 | 1.049 | 1.101 |
| NLR-TyG-AF | Original model | Outcome model | Exposure | NLR | 1.494 | 1 | 1.222 | 1.494 |
| NLR-TyG-AF | Original model | Outcome model | Mediator | TyG | 1.543 | 1 | 1.242 | 1.543 |
| NLR-TyG-AF | Original model | Outcome model | Covariate | Gender | 1.065 | 1 | 1.032 | 1.065 |
| NLR-TyG-AF | Original model | Outcome model | Covariate | Chronic_Kidney_Disease | 1.040 | 1 | 1.020 | 1.040 |
| NLR-TyG-AF | Original model | Outcome model | Covariate | Bradycardia | 1.116 | 1 | 1.057 | 1.116 |
| NLR-TyG-AF | Original model | Outcome model | Covariate | Arrhythmia | 1.181 | 1 | 1.087 | 1.181 |
| NLR-TyG-AF | Original model | Outcome model | Covariate | Anxiety | 1.246 | 1 | 1.116 | 1.246 |
| NLR-TyG-AF | Original model | Outcome model | Covariate | Depression | 1.224 | 1 | 1.106 | 1.224 |
| NLR-TyG-AF | Original model | Outcome model | Covariate | Tumor | 1.051 | 1 | 1.025 | 1.051 |
| NLR-TyG-AF | Original model | Outcome model | Covariate | Hypertension | 1.051 | 1 | 1.025 | 1.051 |
| NLR-TyG-AF | Original model | Outcome model | Covariate | Coronary_Heart_Disease | 1.088 | 1 | 1.043 | 1.088 |
| NLR-TyG-AF | Original model | Outcome model | Covariate | Diabetes | 1.186 | 1 | 1.089 | 1.186 |
| NLR-TyG-AF | Original model | Outcome model | Covariate | Stroke | 1.197 | 1 | 1.094 | 1.197 |
| NLR-TyG-AF | Original model | Outcome model | Covariate | COPD | 1.007 | 1 | 1.004 | 1.007 |
| NLR-TyG-AF | Original model | Outcome model | Covariate | Pneumonia | 1.064 | 1 | 1.031 | 1.064 |
| NLR-TyG-AF | Original model | Outcome model | Covariate | Cirrhosis | 1.006 | 1 | 1.003 | 1.006 |
| NLR-TyG-AF | Original model | Outcome model | Covariate | Rheumatoid_Arthritis | 1.057 | 1 | 1.028 | 1.057 |
| NLR-TyG-AF | Original model | Outcome model | Covariate | Asthma | 1.012 | 1 | 1.006 | 1.012 |
| NLR-TyG-AF | Original model | Outcome model | Covariate | Thyroid_Disease | 1.200 | 1 | 1.095 | 1.200 |
| NLR-TyG-AF | Original model | Outcome model | Covariate | Osteoporosis | 1.118 | 1 | 1.057 | 1.118 |
| NLR-TyG-AF | Original model | Outcome model | Covariate | Anemia | 1.099 | 1 | 1.048 | 1.099 |
| TyG-NLR-AF | Expanded model | Mediator model | Exposure | TyG | 1.151 | 1 | 1.073 | 1.151 |
| TyG-NLR-AF | Expanded model | Mediator model | Covariate | Gender | 1.192 | 1 | 1.092 | 1.192 |
| TyG-NLR-AF | Expanded model | Mediator model | Covariate | Chronic_Kidney_Disease | 1.041 | 1 | 1.020 | 1.041 |
| TyG-NLR-AF | Expanded model | Mediator model | Covariate | Bradycardia | 1.175 | 1 | 1.084 | 1.175 |
| TyG-NLR-AF | Expanded model | Mediator model | Covariate | Arrhythmia | 1.199 | 1 | 1.095 | 1.199 |
| TyG-NLR-AF | Expanded model | Mediator model | Covariate | Anxiety | 1.271 | 1 | 1.128 | 1.271 |
| TyG-NLR-AF | Expanded model | Mediator model | Covariate | Depression | 1.242 | 1 | 1.114 | 1.242 |
| TyG-NLR-AF | Expanded model | Mediator model | Covariate | Tumor | 1.051 | 1 | 1.025 | 1.051 |
| TyG-NLR-AF | Expanded model | Mediator model | Covariate | Hypertension | 1.125 | 1 | 1.061 | 1.125 |
| TyG-NLR-AF | Expanded model | Mediator model | Covariate | Coronary_Heart_Disease | 1.169 | 1 | 1.081 | 1.169 |
| TyG-NLR-AF | Expanded model | Mediator model | Covariate | Diabetes | 1.201 | 1 | 1.096 | 1.201 |
| TyG-NLR-AF | Expanded model | Mediator model | Covariate | Stroke | 1.201 | 1 | 1.096 | 1.201 |
| TyG-NLR-AF | Expanded model | Mediator model | Covariate | COPD | 1.007 | 1 | 1.004 | 1.007 |
| TyG-NLR-AF | Expanded model | Mediator model | Covariate | Pneumonia | 1.124 | 1 | 1.060 | 1.124 |
| TyG-NLR-AF | Expanded model | Mediator model | Covariate | Cirrhosis | 1.010 | 1 | 1.005 | 1.010 |
| TyG-NLR-AF | Expanded model | Mediator model | Covariate | Rheumatoid_Arthritis | 1.063 | 1 | 1.031 | 1.063 |
| TyG-NLR-AF | Expanded model | Mediator model | Covariate | Asthma | 1.013 | 1 | 1.006 | 1.013 |
| TyG-NLR-AF | Expanded model | Mediator model | Covariate | Thyroid_Disease | 1.208 | 1 | 1.099 | 1.208 |
| TyG-NLR-AF | Expanded model | Mediator model | Covariate | Osteoporosis | 1.124 | 1 | 1.060 | 1.124 |
| TyG-NLR-AF | Expanded model | Mediator model | Covariate | Anemia | 1.309 | 1 | 1.144 | 1.309 |
| TyG-NLR-AF | Expanded model | Mediator model | Covariate | Age | 1.343 | 1 | 1.159 | 1.343 |
| TyG-NLR-AF | Expanded model | Mediator model | Covariate | Temperature | 1.006 | 1 | 1.003 | 1.006 |
| TyG-NLR-AF | Expanded model | Mediator model | Covariate | HR | 1.190 | 1 | 1.091 | 1.190 |
| TyG-NLR-AF | Expanded model | Mediator model | Covariate | Respiratory_Rate | 1.024 | 1 | 1.012 | 1.024 |
| TyG-NLR-AF | Expanded model | Mediator model | Covariate | SBP | 1.534 | 1 | 1.239 | 1.534 |
| TyG-NLR-AF | Expanded model | Mediator model | Covariate | DBP | 1.716 | 1 | 1.310 | 1.716 |
| TyG-NLR-AF | Expanded model | Mediator model | Covariate | LDH | 1.072 | 1 | 1.035 | 1.072 |
| TyG-NLR-AF | Expanded model | Mediator model | Covariate | Hemoglobin | 1.475 | 1 | 1.214 | 1.475 |
| TyG-NLR-AF | Expanded model | Mediator model | Covariate | FDP | 1.178 | 1 | 1.085 | 1.178 |
| TyG-NLR-AF | Expanded model | Mediator model | Covariate | Magnesium | 1.047 | 1 | 1.023 | 1.047 |
| TyG-NLR-AF | Expanded model | Outcome model | Exposure | TyG | 1.608 | 1 | 1.268 | 1.608 |
| TyG-NLR-AF | Expanded model | Outcome model | Mediator | NLR | 1.549 | 1 | 1.245 | 1.549 |
| TyG-NLR-AF | Expanded model | Outcome model | Covariate | Gender | 1.185 | 1 | 1.089 | 1.185 |
| TyG-NLR-AF | Expanded model | Outcome model | Covariate | Chronic_Kidney_Disease | 1.045 | 1 | 1.022 | 1.045 |
| TyG-NLR-AF | Expanded model | Outcome model | Covariate | Bradycardia | 1.160 | 1 | 1.077 | 1.160 |
| TyG-NLR-AF | Expanded model | Outcome model | Covariate | Arrhythmia | 1.192 | 1 | 1.092 | 1.192 |
| TyG-NLR-AF | Expanded model | Outcome model | Covariate | Anxiety | 1.247 | 1 | 1.117 | 1.247 |
| TyG-NLR-AF | Expanded model | Outcome model | Covariate | Depression | 1.220 | 1 | 1.105 | 1.220 |
| TyG-NLR-AF | Expanded model | Outcome model | Covariate | Tumor | 1.052 | 1 | 1.026 | 1.052 |
| TyG-NLR-AF | Expanded model | Outcome model | Covariate | Hypertension | 1.128 | 1 | 1.062 | 1.128 |
| TyG-NLR-AF | Expanded model | Outcome model | Covariate | Coronary_Heart_Disease | 1.160 | 1 | 1.077 | 1.160 |
| TyG-NLR-AF | Expanded model | Outcome model | Covariate | Diabetes | 1.218 | 1 | 1.104 | 1.218 |
| TyG-NLR-AF | Expanded model | Outcome model | Covariate | Stroke | 1.227 | 1 | 1.108 | 1.227 |
| TyG-NLR-AF | Expanded model | Outcome model | Covariate | COPD | 1.009 | 1 | 1.005 | 1.009 |
| TyG-NLR-AF | Expanded model | Outcome model | Covariate | Pneumonia | 1.121 | 1 | 1.059 | 1.121 |
| TyG-NLR-AF | Expanded model | Outcome model | Covariate | Cirrhosis | 1.009 | 1 | 1.005 | 1.009 |
| TyG-NLR-AF | Expanded model | Outcome model | Covariate | Rheumatoid_Arthritis | 1.065 | 1 | 1.032 | 1.065 |
| TyG-NLR-AF | Expanded model | Outcome model | Covariate | Asthma | 1.013 | 1 | 1.006 | 1.013 |
| TyG-NLR-AF | Expanded model | Outcome model | Covariate | Thyroid_Disease | 1.214 | 1 | 1.102 | 1.214 |
| TyG-NLR-AF | Expanded model | Outcome model | Covariate | Osteoporosis | 1.126 | 1 | 1.061 | 1.126 |
| TyG-NLR-AF | Expanded model | Outcome model | Covariate | Anemia | 1.341 | 1 | 1.158 | 1.341 |
| TyG-NLR-AF | Expanded model | Outcome model | Covariate | Age | 1.309 | 1 | 1.144 | 1.309 |
| TyG-NLR-AF | Expanded model | Outcome model | Covariate | Temperature | 1.006 | 1 | 1.003 | 1.006 |
| TyG-NLR-AF | Expanded model | Outcome model | Covariate | HR | 1.186 | 1 | 1.089 | 1.186 |
| TyG-NLR-AF | Expanded model | Outcome model | Covariate | Respiratory_Rate | 1.023 | 1 | 1.012 | 1.023 |
| TyG-NLR-AF | Expanded model | Outcome model | Covariate | SBP | 1.555 | 1 | 1.247 | 1.555 |
| TyG-NLR-AF | Expanded model | Outcome model | Covariate | DBP | 1.740 | 1 | 1.319 | 1.740 |
| TyG-NLR-AF | Expanded model | Outcome model | Covariate | LDH | 1.075 | 1 | 1.037 | 1.075 |
| TyG-NLR-AF | Expanded model | Outcome model | Covariate | Hemoglobin | 1.497 | 1 | 1.223 | 1.497 |
| TyG-NLR-AF | Expanded model | Outcome model | Covariate | FDP | 1.202 | 1 | 1.096 | 1.202 |
| TyG-NLR-AF | Expanded model | Outcome model | Covariate | Magnesium | 1.057 | 1 | 1.028 | 1.057 |
| NLR-TyG-AF | Expanded model | Mediator model | Exposure | NLR | 1.120 | 1 | 1.058 | 1.120 |
| NLR-TyG-AF | Expanded model | Mediator model | Covariate | Gender | 1.197 | 1 | 1.094 | 1.197 |
| NLR-TyG-AF | Expanded model | Mediator model | Covariate | Chronic_Kidney_Disease | 1.041 | 1 | 1.020 | 1.041 |
| NLR-TyG-AF | Expanded model | Mediator model | Covariate | Bradycardia | 1.175 | 1 | 1.084 | 1.175 |
| NLR-TyG-AF | Expanded model | Mediator model | Covariate | Arrhythmia | 1.198 | 1 | 1.095 | 1.198 |
| NLR-TyG-AF | Expanded model | Mediator model | Covariate | Anxiety | 1.271 | 1 | 1.127 | 1.271 |
| NLR-TyG-AF | Expanded model | Mediator model | Covariate | Depression | 1.241 | 1 | 1.114 | 1.241 |
| NLR-TyG-AF | Expanded model | Mediator model | Covariate | Tumor | 1.052 | 1 | 1.026 | 1.052 |
| NLR-TyG-AF | Expanded model | Mediator model | Covariate | Hypertension | 1.124 | 1 | 1.060 | 1.124 |
| NLR-TyG-AF | Expanded model | Mediator model | Covariate | Coronary_Heart_Disease | 1.167 | 1 | 1.080 | 1.167 |
| NLR-TyG-AF | Expanded model | Mediator model | Covariate | Diabetes | 1.175 | 1 | 1.084 | 1.175 |
| NLR-TyG-AF | Expanded model | Mediator model | Covariate | Stroke | 1.195 | 1 | 1.093 | 1.195 |
| NLR-TyG-AF | Expanded model | Mediator model | Covariate | COPD | 1.007 | 1 | 1.003 | 1.007 |
| NLR-TyG-AF | Expanded model | Mediator model | Covariate | Pneumonia | 1.126 | 1 | 1.061 | 1.126 |
| NLR-TyG-AF | Expanded model | Mediator model | Covariate | Cirrhosis | 1.009 | 1 | 1.004 | 1.009 |
| NLR-TyG-AF | Expanded model | Mediator model | Covariate | Rheumatoid_Arthritis | 1.062 | 1 | 1.031 | 1.062 |
| NLR-TyG-AF | Expanded model | Mediator model | Covariate | Asthma | 1.013 | 1 | 1.007 | 1.013 |
| NLR-TyG-AF | Expanded model | Mediator model | Covariate | Thyroid_Disease | 1.202 | 1 | 1.096 | 1.202 |
| NLR-TyG-AF | Expanded model | Mediator model | Covariate | Osteoporosis | 1.123 | 1 | 1.060 | 1.123 |
| NLR-TyG-AF | Expanded model | Mediator model | Covariate | Anemia | 1.308 | 1 | 1.144 | 1.308 |
| NLR-TyG-AF | Expanded model | Mediator model | Covariate | Age | 1.343 | 1 | 1.159 | 1.343 |
| NLR-TyG-AF | Expanded model | Mediator model | Covariate | Temperature | 1.006 | 1 | 1.003 | 1.006 |
| NLR-TyG-AF | Expanded model | Mediator model | Covariate | HR | 1.191 | 1 | 1.091 | 1.191 |
| NLR-TyG-AF | Expanded model | Mediator model | Covariate | Respiratory_Rate | 1.023 | 1 | 1.011 | 1.023 |
| NLR-TyG-AF | Expanded model | Mediator model | Covariate | SBP | 1.534 | 1 | 1.239 | 1.534 |
| NLR-TyG-AF | Expanded model | Mediator model | Covariate | DBP | 1.716 | 1 | 1.310 | 1.716 |
| NLR-TyG-AF | Expanded model | Mediator model | Covariate | LDH | 1.076 | 1 | 1.038 | 1.076 |
| NLR-TyG-AF | Expanded model | Mediator model | Covariate | Hemoglobin | 1.431 | 1 | 1.196 | 1.431 |
| NLR-TyG-AF | Expanded model | Mediator model | Covariate | FDP | 1.196 | 1 | 1.094 | 1.196 |
| NLR-TyG-AF | Expanded model | Mediator model | Covariate | Magnesium | 1.046 | 1 | 1.023 | 1.046 |
| NLR-TyG-AF | Expanded model | Outcome model | Exposure | NLR | 1.549 | 1 | 1.245 | 1.549 |
| NLR-TyG-AF | Expanded model | Outcome model | Mediator | TyG | 1.608 | 1 | 1.268 | 1.608 |
| NLR-TyG-AF | Expanded model | Outcome model | Covariate | Gender | 1.185 | 1 | 1.089 | 1.185 |
| NLR-TyG-AF | Expanded model | Outcome model | Covariate | Chronic_Kidney_Disease | 1.045 | 1 | 1.022 | 1.045 |
| NLR-TyG-AF | Expanded model | Outcome model | Covariate | Bradycardia | 1.160 | 1 | 1.077 | 1.160 |
| NLR-TyG-AF | Expanded model | Outcome model | Covariate | Arrhythmia | 1.192 | 1 | 1.092 | 1.192 |
| NLR-TyG-AF | Expanded model | Outcome model | Covariate | Anxiety | 1.247 | 1 | 1.117 | 1.247 |
| NLR-TyG-AF | Expanded model | Outcome model | Covariate | Depression | 1.220 | 1 | 1.105 | 1.220 |
| NLR-TyG-AF | Expanded model | Outcome model | Covariate | Tumor | 1.052 | 1 | 1.026 | 1.052 |
| NLR-TyG-AF | Expanded model | Outcome model | Covariate | Hypertension | 1.128 | 1 | 1.062 | 1.128 |
| NLR-TyG-AF | Expanded model | Outcome model | Covariate | Coronary_Heart_Disease | 1.160 | 1 | 1.077 | 1.160 |
| NLR-TyG-AF | Expanded model | Outcome model | Covariate | Diabetes | 1.218 | 1 | 1.104 | 1.218 |
| NLR-TyG-AF | Expanded model | Outcome model | Covariate | Stroke | 1.227 | 1 | 1.108 | 1.227 |
| NLR-TyG-AF | Expanded model | Outcome model | Covariate | COPD | 1.009 | 1 | 1.005 | 1.009 |
| NLR-TyG-AF | Expanded model | Outcome model | Covariate | Pneumonia | 1.121 | 1 | 1.059 | 1.121 |
| NLR-TyG-AF | Expanded model | Outcome model | Covariate | Cirrhosis | 1.009 | 1 | 1.005 | 1.009 |
| NLR-TyG-AF | Expanded model | Outcome model | Covariate | Rheumatoid_Arthritis | 1.065 | 1 | 1.032 | 1.065 |
| NLR-TyG-AF | Expanded model | Outcome model | Covariate | Asthma | 1.013 | 1 | 1.006 | 1.013 |
| NLR-TyG-AF | Expanded model | Outcome model | Covariate | Thyroid_Disease | 1.214 | 1 | 1.102 | 1.214 |
| NLR-TyG-AF | Expanded model | Outcome model | Covariate | Osteoporosis | 1.126 | 1 | 1.061 | 1.126 |
| NLR-TyG-AF | Expanded model | Outcome model | Covariate | Anemia | 1.341 | 1 | 1.158 | 1.341 |
| NLR-TyG-AF | Expanded model | Outcome model | Covariate | Age | 1.309 | 1 | 1.144 | 1.309 |
| NLR-TyG-AF | Expanded model | Outcome model | Covariate | Temperature | 1.006 | 1 | 1.003 | 1.006 |
| NLR-TyG-AF | Expanded model | Outcome model | Covariate | HR | 1.186 | 1 | 1.089 | 1.186 |
| NLR-TyG-AF | Expanded model | Outcome model | Covariate | Respiratory_Rate | 1.023 | 1 | 1.012 | 1.023 |
| NLR-TyG-AF | Expanded model | Outcome model | Covariate | SBP | 1.555 | 1 | 1.247 | 1.555 |
| NLR-TyG-AF | Expanded model | Outcome model | Covariate | DBP | 1.740 | 1 | 1.319 | 1.740 |
| NLR-TyG-AF | Expanded model | Outcome model | Covariate | LDH | 1.075 | 1 | 1.037 | 1.075 |
| NLR-TyG-AF | Expanded model | Outcome model | Covariate | Hemoglobin | 1.497 | 1 | 1.223 | 1.497 |
| NLR-TyG-AF | Expanded model | Outcome model | Covariate | FDP | 1.202 | 1 | 1.096 | 1.202 |
| NLR-TyG-AF | Expanded model | Outcome model | Covariate | Magnesium | 1.057 | 1 | 1.028 | 1.057 |
| VIF indicates variance inflation factor; GVIF, generalized variance inflation factor. For terms with more than one degree of freedom, GVIF^(1/[2 x Df]) is reported as the adjusted GVIF. Comparable VIF was calculated as the square of the adjusted GVIF so that it can be interpreted on the usual VIF scale. The mediator model includes the exposure and covariates, whereas the outcome model simultaneously includes the exposure, mediator, and covariates. | | | | | | | | |

# Supplementary Figures

## eFig 1 Patient screening flowchart

## eFig 2 Feature selection using least absolute shrinkage and selection operator (LASSO) regression and the Boruta algorithm

**(A)** Five-fold cross-validation curve for LASSO regression. **(B)** LASSO coefficient profiles, with the upper axis indicating the number of nonzero coefficients. **(C)** Boruta importance distributions for the 30 LASSO-selected variables. Red, yellow, green, and blue indicate confirmed, tentative, rejected, and shadow features, respectively; asterisks mark the six variables retained by Boruta.

## eFig 3 Comparison of the decision curve analysis and calibration performance of 9 machine learning models

(A) Decision curve analysis of the training set. (B) Decision curve analysis of the internal test set. (C) Decision curve analysis of the external validation set. (D) Calibration curves of the training set. (E) Calibration curves of the internal test set. (F) Calibration curves of the external validation set.

## eFig 4 Exploratory mediation analysis of the bidirectional associations among NLR, TyG, and recent-onset atrial fibrillation

**Abbreviations:** ACME, average causal mediation effect; ADE, average direct effect; AF, recent-onset atrial fibrillation; NLR, neutrophil-to-lymphocyte ratio; TyG, triglyceride-glucose index.

## eFig 5 Estimated proportions mediated in the bidirectional exploratory mediation analyses of NLR, TyG, and recent-onset atrial fibrillation

**Abbreviations:** AF, recent-onset atrial fibrillation; NLR, neutrophil-to-lymphocyte ratio; TyG, triglyceride-glucose index.
